# Auxin biosynthesis and signaling drive virulence and plant adaptation in *Dickeya dadantii*

**DOI:** 10.1371/journal.ppat.1014429

**Published:** 2026-07-17

**Authors:** Amalia Roca, Saray Santamaría-Hernando, Zulema Udaondo, Juan J. Cabrera, Patricia Godoy, Ana Nogueira, Emilia López-Solanilla, Miguel A. Matilla

**Affiliations:** 1 Department of Microbiology, Facultad de Farmacia, Campus Universitario de Cartuja, Universidad de Granada, Granada, Spain; 2 Institute of Biotechnology, Biomedical Research Center (CIBM), University of Granada, Granada, Spain; 3 Centro de Biotecnología y Genómica de Plantas, Universidad Politécnica de Madrid (UPM)-Instituto Nacional de Investigación y Tecnología Agraria y Alimentaria (INIA-CSIC), Pozuelo de Alarcón, Madrid, Spain; 4 Department of Biotechnology-Plant Biology, Escuela Técnica Superior de Ingeniería Agronómica, Alimentaria y de Biosistemas, Universidad Politécnica de Madrid (UPM), Madrid, Spain; 5 Department of Microbial Biotechnology, Centro Nacional de Biotecnología, Consejo Superior de Investigaciones Científicas, Madrid, Spain; 6 Department of Biotechnology and Environmental Protection, Estación Experimental del Zaidín, Consejo Superior de Investigaciones Científicas, Granada, Spain; University of Florida Institute of Food and Agricultural Sciences, UNITED STATES OF AMERICA

## Abstract

Plants and their associated bacteria engage in complex bidirectional interactions mediated by a diverse array of signaling molecules of both plant and microbial origin. Indole-3-acetic acid (IAA) is a central phytohormone regulating plant growth and development and is increasingly recognized as an intra- and inter-kingdom signaling molecule that modulates diverse bacterial processes relevant during plant–microbe interactions. While IAA biosynthesis is widespread among plant-associated bacteria, the mechanisms through which this auxin regulates bacterial physiology and virulence, as well as those controlling its production, remain poorly understood. Here, we show that IAA synthesis deficiency in the global phytopathogen *Dickeya dadantii* triggers transcriptional reprogramming and results in reduced virulence and fitness during plant infection. Endogenous IAA was found to regulate the expression of the AaeXAB efflux pump, which mediates endogenous IAA secretion, confers resistance to plant defense–related phytohormones, and contributes to plant virulence in *D. dadantii*. Consistent with its role in plant-bacteria interactions, phylogenetic analyses revealed that AaeXAB-encoding genes are commonly present among Pseudomonadota isolated from plant-associated environments. Moreover, IAA deficiency altered the expression of a previously uncharacterized indole-responsive MarR-type regulator, DDA3937_RS07305, suggesting cross-talk between IAA- and indole-mediated signaling networks. Our data also uncover a complex regulatory circuit coordinating IAA production in *D. dadantii*, involving the ExpIR and Vfm quorum-sensing systems and the transcriptional regulators TyrR, TrpR, and LrhA. Collectively, our findings provide new insights into the role of IAA as a bacterial signal promoting plant adaptation and virulence. Targeting IAA biosynthesis and efflux pump activity may offer promising avenues for the development of anti-virulence strategies in phytopathogenesis.

## Author summary

Plants live in close association with large bacterial communities. Plant-bacteria interactions rely on a complex chemical language exchanged between both partners. One of these signal molecules is indole-3-acetic acid (IAA), a plant hormone best known for controlling plant growth and development, which is also produced by many plant-associated bacteria. Here, we investigated how IAA influences bacterial behavior using *Dickeya dadantii* - a globally important plant pathogen that causes soft-rot disease in major crops such as potato, maize and carrot. We show that deficiency in IAA production reduces the ability of this bacterium to infect plants and to compete successfully within plant tissues. Loss of IAA production also leads to changes in gene expression, affecting traits important for survival and disease development. These include a transport system that promotes IAA export in *D. dadantii* and protects the bacterium against plant defense compounds. Homologous transport systems are widespread among plant-associated bacteria, suggesting a broader biological relevance. Our work further reveals that IAA production is tightly controlled by multiple regulatory systems. Together, our findings identify IAA as a key bacterial signal that promotes adaptation to plant hosts and disease development. Understanding these processes may help guide new strategies for controlling plant diseases.

## Introduction

Indole-3-acetic acid (IAA) was the first phytohormone identified and remains the most abundant naturally occurring auxin in plants [1,2]. Its origin and functional diversification are thought to have coincided with plant terrestrialization approximately 480 million years ago [3]. IAA functions as a central plant signaling molecule that orchestrates growth, development, and stress resistance [1–6], with highly concentration-dependent activity that is tightly regulated through control of its biosynthesis, transport, and inactivation [2,7]. Notably, IAA biosynthesis is not restricted to plants but occurs broadly across the Tree of Life [3,8–12]. In bacteria, IAA production is widespread, with over 80% of species estimated to possess this capacity [13]. Bacterial IAA biosynthesis proceeds through tryptophan-dependent and tryptophan-independent pathways, depending on whether L-tryptophan (L-Trp) serves as the precursor [14–16]. Analogously to plants [1,7], five tryptophan-dependent pathways have been described in bacteria, each characterized by distinct biosynthetic intermediates: indole-3-acetamide (IAM), indole-3-pyruvic acid (IPA), indole-3-acetonitrile (IAN), tryptamine (TPM), and L-Trp side-chain oxidase (TSO) pathways [14–16]. Alternatively, microbial tryptophan-independent pathways typically employ indole or indole-3-glycerol phosphate as precursors [15,16]. A single bacterial species often harbors multiple IAA biosynthetic routes [13,16], highlighting the biological importance and evolutionary conservation of IAA metabolism in bacteria.

IAA biosynthesis is prevalent among plant-associated bacteria (PAB), including both beneficial and pathogenic species [5,10,13,15,17] – supporting its central role as an inter-kingdom signal molecule in plant-microbe interactions. In plant growth-promoting bacteria, IAA production typically enhances plant growth by modulating root development and architecture, enhancing nutrient uptake, and increasing stress tolerance [10,15,18,19]. However, plants tightly regulate endogenous IAA levels through multiple mechanisms that ensure proper auxin homeostasis [2,7], and bacterial IAA production can lead to supraoptimal concentrations that negatively affect plant growth [20] and health [5,15,21]. Specifically, this strategy is exploited by phytopathogenic bacteria, as accumulating evidence implicates IAA biosynthesis in virulence through several independent mechanisms, including: (i) regulation of the expression of virulence determinants [22–24]; (ii) alteration of host signaling and physiology to suppress plant defenses and promote disease development; and (iii) induction of uncontrolled plant cell proliferation, as exemplified by IAA production in tumorigenic crop pathogens such as *Pseudomonas savastanoi* and *Pantoea agglomerans*, or by auxin synthesis induced in plants by the gall-inducing phytopathogen *Agrobacterium tumefaciens* following T-DNA integration into the plant genome [5,15,21]. Among PAB, the IAM, IPA, and TPM pathways are the most prevalent routes for IAA biosynthesis [13,15,16], with the IAM pathway being particularly associated with phytopathogenicity [13,16,21].

Beyond its role as an inter-kingdom signal that modulates plant growth and disease development, IAA is also emerging as an important bacterial signal molecule that regulates gene expression and multiple physiological processes relevant to plant-microbe interactions. Among these, IAA has been shown to regulate motility [23,25,26], biofilm formation [25,27], chemotaxis [23,28], stress resistance [23,25,27,29], efflux pumps [25], secretion systems [22,23,25,30], antimicrobial compound production [31–33], nodulation [27,34], nitrogen fixation [27,29,34], metabolism [25,27,30,35–37], and the expression of virulence genes during infection [5,21,23,38,39]. Despite this functional diversity, the molecular mechanisms by which IAA mediates such broad regulatory effects remain poorly understood. Moreover, given the wide range of processes modulated by IAA, its biosynthesis is tightly controlled at both the transcriptional [14,15,36,40,41] and post-transcriptional [42,43] levels. However, the regulatory mechanisms governing IAA synthesis, as well as the signals that modulate its production, remain poorly characterized and represent a major gap in current knowledge.

*Dickeya dadantii* (formerly *Erwinia chrysanthemi*) is among the most significant bacterial plant pathogens [44]. Within the *Dickeya* genus, it exhibits one of the broadest host ranges, infecting and causing soft rot disease in a wide array of economically important crops and ornamental plants [45]. *Dickeya dadantii* 3937 serves as a model strain for studying the mechanisms of phytopathogenesis within the soft rot *Pectobacteriaceae* [45,46]. This strain synthesizes IAA via the IAM pathway through two sequential enzymatic reactions catalyzed by the tryptophan-2-monooxygenase IaaM and the indole-3-acetamide hydrolase IaaH [15,47]. Previous studies revealed that IAA biosynthetic genes in *D. dadantii* 3937 are upregulated during host plant colonization [48] and subsequent work supported a role for IAA as a signaling molecule in this phytopathogen [47].

In this study, we employ a multidisciplinary approach to investigate the mechanisms underlying IAA function as an endogenous signal molecule in *D. dadantii* and to explore its contribution to virulence and colonization fitness *in planta*. We show that endogenous IAA production regulates the expression of an efflux pump involved in auxin secretion, plant virulence, and resistance to elevated levels of IAA and other plant signaling molecules, representing the first IAA efflux pump described in Soft Rot *Pectobacteriaceae.* We also analyze the phylogenetic distribution of this efflux system, highlighting that it is widely present in plant-associated Pseudomonadota. In addition, we explore the regulatory networks governing IAA biosynthesis in *D. dadantii*. Collectively, our findings highlight the pivotal role of IAA biosynthesis in plant host adaptation and virulence, and uncover a complex regulatory network involving multiple quorum-sensing systems and transcriptional regulators that coordinate auxin production in this globally significant phytopathogen.

## Results

### Transcriptomic effects of IAA biosynthesis deficiency in *D. dadantii*

**Fig 1:**
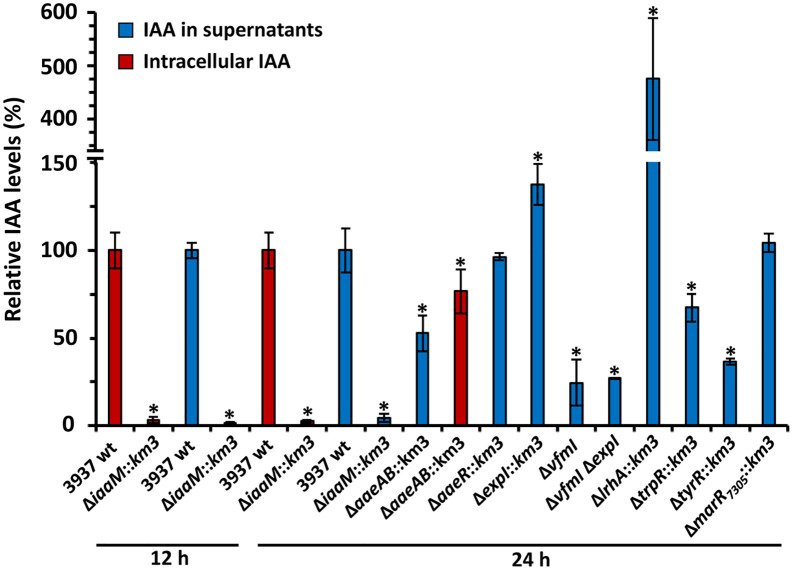
Quantification of indole-3-acetic acid (IAA) production by *Dickeya dadantii* 3937 strains measured by gas chromatography coupled to mass spectrometry. Shown are the relative IAA levels in supernatants and cell lysates of different *D. dadantii* 3937 strains grown at 28 °C for 12 and 24 h in minimal medium supplemented with 0.25 mg/mL L-Trp. Data represent means and standard deviations of three biological replicates. *p < 0.01 (Student's *t*-test) comparing mutant strains to the 3937 wild-type strain. IaaM, tryptophan-2-monooxygenase involved in IAA biosynthesis; AaeAB, subunits A and B of the AaeXAB efflux system; AaeR, LysR-type transcriptional regulator associated with the *aaeXAB* genes; ExpI, acyl-homoserine-lactone synthase; VfmI, histidine kinase involved in signal perception and activation of the Vfm quorum-sensing system; LrhA, LysR-type transcriptional regulator; TrpR, tryptophan operon repressor; TyrR, transcriptional regulator; MarR_7305_, MarR-type transcriptional regulator DDA3937_RS07305.

To investigate the role of endogenous IAA as a signaling molecule in *D. dadantii* 3937, we first constructed an *iaaM* mutant strain, deficient in the key enzyme that converts L-Trp into the indole-3-acetamide intermediate of the IAM biosynthetic pathway [15]. IAA levels in culture supernatants from the wild-type and *iaaM* mutant strains were then quantified by gas chromatography–mass spectrometry (GC-MS). The *iaaM* mutant exhibited a drastic reduction in IAA production, corresponding to only 1.7% and 4.6% of wild-type levels after 12 h and 24 h of growth, respectively (Fig 1). Similarly, intracellular IAA concentrations in the mutant were 3.4% and 2.6% of those in the wild-type strain at the same time points (Fig 1). Expression of the *iaaM* gene *in trans* from a pBBR-based plasmid not only restored IAA production in the *iaaM* mutant strain but resulted in auxin overproduction (Fig A in S1 Text).

**Fig 2:**
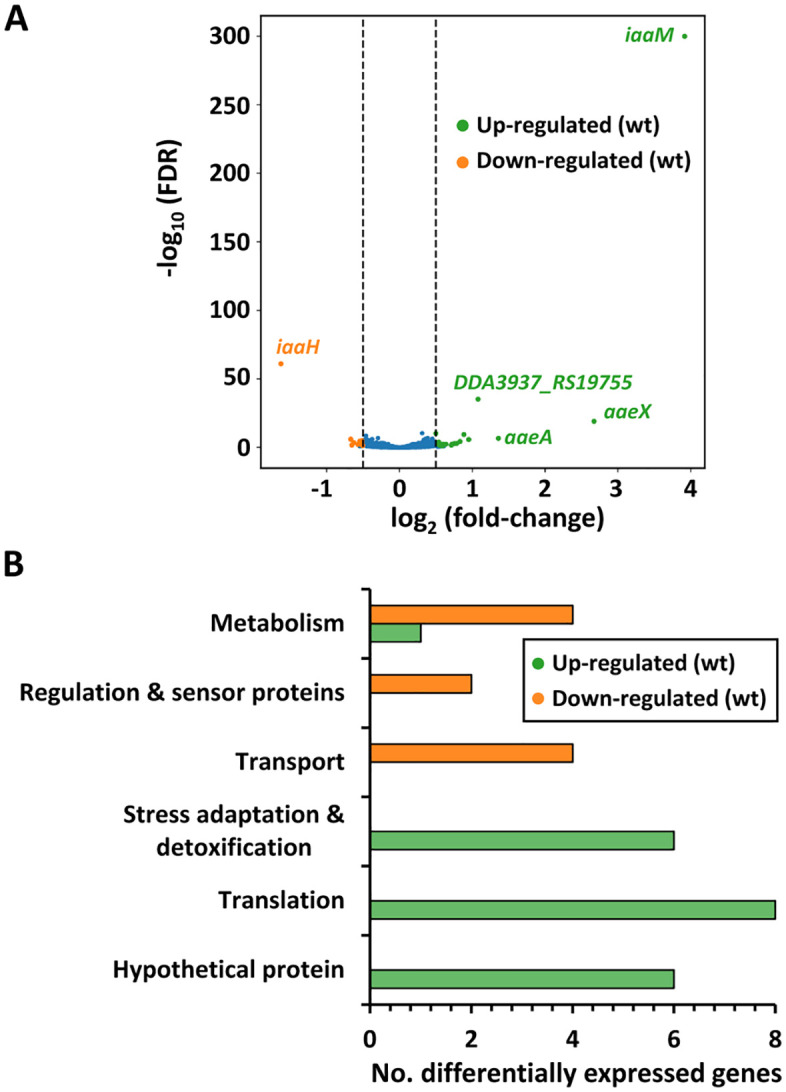
RNA-seq analysis comparing the wild-type and *iaaM* mutant strains of *D. dadantii* 3937. A, Volcano plot depicting differentially expressed genes in the wild-type relative to *iaaM* mutant. The x-axis represents the log_2_ fold change, and the y-axis shows the statistical significance (−log_10_ of the adjusted *p*-value (*p*adj) <0.05) for each gene. Vertical dashed lines indicate the Log_2_FC threshold of ≥|0.5|. Blue dots denote genes that were not significantly differentially expressed. B, Functional categorization of the significantly differentially expressed genes.

Given these remarkable differences in IAA biosynthesis, we next compared the global transcriptomes of the wild-type and *iaaM*-deficient strains. Because IAA production in *D. dadantii* 3937 begins during exponential growth and peaks in early stationary phase [49], total RNA was extracted from cultures grown in minimal medium and collected at early stationary phase, corresponding to 12 h of growth. Quantitative GC–MS analysis of the supernatants of these cultures revealed IAA concentrations of 86.6 ± 3.8 µM in the wild-type and 1.5 ± 0.3 µM in the *iaaM* mutant. Additional measurements at 24 h showed IAA concentrations of 160 ± 12.2 μM and 6.5 ± 2.5 μM in the wild-type and *iaaM* mutant strains, respectively. RNA-seq analysis identified 31 differentially expressed genes (DEGs), defined by an absolute log₂ fold change greater than 0.5 and an adjusted *p*-value (*p*adj) below 0.05, using the Benjamini–Hochberg correction (Fig 2 and Table 1). This represents approximately 0.7% of the total *D. dadantii* 3937 genes [50]. Among these, 21 genes were upregulated and 10 were downregulated in the wild-type strain (Fig 2 and Table 1). These DEGs were functionally categorized into metabolism (16%), regulation and sensing (6.5%), transport (13%), stress adaptation and detoxification (19%), and translation (26%), with 19% encoding proteins of unknown function (Fig 2B and Table 1). The most enriched categories in the wild-type strain were stress adaptation and detoxification, and translation, whereas genes related to transport and metabolism were more highly expressed in the *iaaM* mutant. These transcriptomic results were validated by quantitative real time PCR (RT-qPCR) for representative genes from various functional categories, showing strong correlation with the RNA-seq data (Fig B in S1 Text).

**Table 1:** Differentially expressed genes in wild-type *Dickeya dadantii* 3937 versus ∆*iaaM*::*km3.*

Locus no.	Gene name	Known or predicted function	Log_2_Fold change^a^	Fold change	KEGG Orthology^b^
**UPREGULATED (in the wild type)**					
**Metabolism**					
DDA3937_RS01905	*iaaM*	Tryptophan-2-monooxygenase	3.91^c^	15.0^c^	AAM
**Stress adaptation & detoxification**					
DDA3937_RS01470	*aaeX*	*p*-hydroxybenzoic acid efflux pump operon protein AaeX	2.67	6.4	SCP
DDA3937_RS01475	*aaeA*	*p*-hydroxybenzoic acid efflux pump subunit AaeA	1.36	2.6	SCP
DDA3937_RS06115	*cspE*	Transcription antiterminator/RNA stability regulator CspE	0.95	1.9	GIP
DDA3937_RS19295	*bfr*	Bacterioferritin	0.5	1.4	MCV
DDA3937_RS19755	*–*	Cold-shock protein	1.08	2.1	GIP
DDA3937_RS22030	–	AcrZ family multidrug efflux pump-associated protein	0.83	1.8	SCP
**Translation**					
DDA3937_RS00065	*–*	tRNA-Glu	0.75	1.7	TL
DDA3937_RS01010	–	tRNA-Glu	0.75	1.7	TL
DDA3937_RS03055	*–*	tRNA-Met	0.89	1.9	TL
DDA3937_RS06530	*–*	tRNA-Lys	0.72	1.7	TL
DDA3937_RS16090	–	tRNA-Glu	0.79	1.7	TL
DDA3937_RS16170	*rimR*	Ribosome maturation factor RimM	0.55	1.5	GIP
DDA3937_RS16215	–	tRNA-Arg	0.71	1.6	TL
DDA3937_RS19190	*rplO*	50S ribosomal protein L15	0.60	1.5	TL
**Unknown function**					
DDA3937_RS04130	*–*	Hypothetical protein	0.54	1.5	Unclassified
DDA3937_RS04440	–	Hypothetical protein	0.55	1.5	Unclassified
DDA3937_RS07580	*–*	Hypothetical protein	0.57	1.5	Unclassified
DDA3937_RS10400	–	Hypothetical protein	0.64	1.6	Unclassified
DDA3937_RS13630	*–*	Hypothetical protein	0.57	1.5	Unclassified
DDA3937_RS23395	–	Hypothetical protein	0.59	1.5	Unclassified
**DOWNREGULATED (in the wild type)**					
**METABOLISM**					
DDA3937_RS01910	*iaaH*	Indole-3-acetamide hydrolase	-1.63	-3.1	AAM
DDA3937_RS11900	*yiaY*	L-threonine dehydrogenase	-0.54	-1.5	AD
DDA3937_RS15785	*grcA*	Autonomous glycyl radical cofactor GrcA	-0.63	-1.5	Unclassified
DDA3937_RS20770	*–*	Vicinal oxygen chelate (VOC) family	-0.52	-1.4	Unclassified
**Transcriptional regulators, regulatory proteins and sensor proteins**					
DDA3937_RS07305	*–*	MarR family transcriptional regulator	-0.57	-1.5	GIP
DDA3937_RS07555	–	GntR family transcriptional regulator	-0.52	-1.4	GIP
**Transport**					
DDA3937_RS06665	*–*	Sugar ABC transporter substrate-binding protein	-0.52	-1.4	T
DDA3937_RS07515	–	ABC transporter permease	-0.51	-1.4	T
DDA3937_RS14490	*–*	Bug family tripartite tricarboxylate transporter substrate binding protein	-0.65	-1.6	T
DDA3937_RS21445	–	ABC transporter substrate-binding protein	-0.67	-1.6	T

^a^Only genes with a Log_2_FC ≥ |0.5| and FDR correction ≤0.05 are listed.

^b^AAM, amino acid metabolism; AD, alcohol dehydrogenase; GIP, genetic information processing; SCP, signaling and cellular processes; T, transport; MCV, metabolism of cofactors and vitamins; TL, translation.

^c^No read coverage was detected across the deleted region of *iaaM* in the mutant samples. The reported fold change value reflects reference-based quantification and downstream normalization/statistical modeling and does not indicate residual expression of the deleted gene.

### The auxin-induced AaeXAB efflux pump mediates IAA export in *D. dadantii*

**Fig 3:**
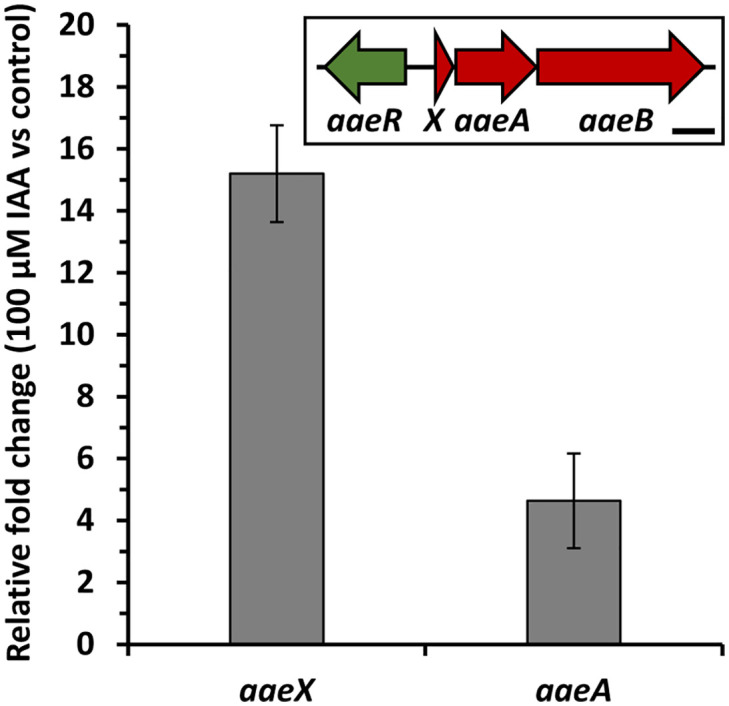
Impact of IAA on the transcript levels of the *aaeXAB* locus in *D. dadantii* 3937. Shown are the fold changes of *aaeX* and *aaeA* mRNA levels in response to 100 µM IAA relative to the untreated control, as measured by quantitative RT-PCR. Samples were collected 30 min after the addition of 100 µM IAA to minimal medium during exponential growth (OD_600_). No IAA was added to control samples. Data represent the means and standard deviations of three biological replicates, each performed in triplicate. The inset panel shows the genetic organization of the chromosomal region containing the *aaeRXAB* genes of *D. dadantii* 3937. Scale bar, 0.5 kbp.

Our transcriptomic analysis revealed that the *aaeX* and *aaeA* genes encoding the AaeXAB efflux pump, firstly characterized in *Escherichia coli* as an aromatic carboxylic acid transporter involved in *p*-hydroxybenzoic acid resistance [51], were among the most strongly upregulated in the wild-type strain (Fig 2A and Table 1) - a result further validated by RT-qPCR (Fig B in S1 Text). Given the markedly reduced intra- and extracellular IAA levels in the *iaaM* mutant (Fig 1), and the induction of the homologous efflux pump by IAA in the rhizobacterium *Serratia plymuthica* A153 [25], these results suggest that IAA positively regulates *aaeXAB* expression in *D. dadantii* 3937. To test this hypothesis, we quantified *aaeX* and *aaeA* transcript levels in cultures grown in the presence and absence of 100 µM IAA – a physiologically relevant concentration comparable to that detected in wild-type culture supernatants, as described above. Exogenous IAA increased *aaeX* and *aaeA* transcript levels by 15.2- and 4.6-fold, respectively (Fig 3), supporting a role for this auxin as an inducer of the efflux pump. We next examined whether the AaeXAB pump contributes to IAA secretion in *D. dadantii* 3937. To this end, we generated a deletion mutant lacking the *aaeAB* genes, which encode subunits A and B of this efflux system [51], and IAA in supernatants of the wild-type and ∆*aaeAB* strains was quantified by GC–MS. The ∆*aaeAB* mutant displayed nearly a 50% reduction in secreted IAA compared to the wild-type (Fig 1), supporting that the AaeXAB pump mediates efflux of endogenously produced IAA. We then determined intracellular IAA levels in the ∆*aaeAB* mutant and observed a ~ 23% reduction compared with the wild-type strain (Fig 1), which may reflect the existence of a regulatory feedback mechanism affecting IAA biosynthesis in response to reduced IAA efflux.

### AaeXAB confers resistance to high IAA and salicylic acid levels in *D. dadantii*

**Fig 4:**
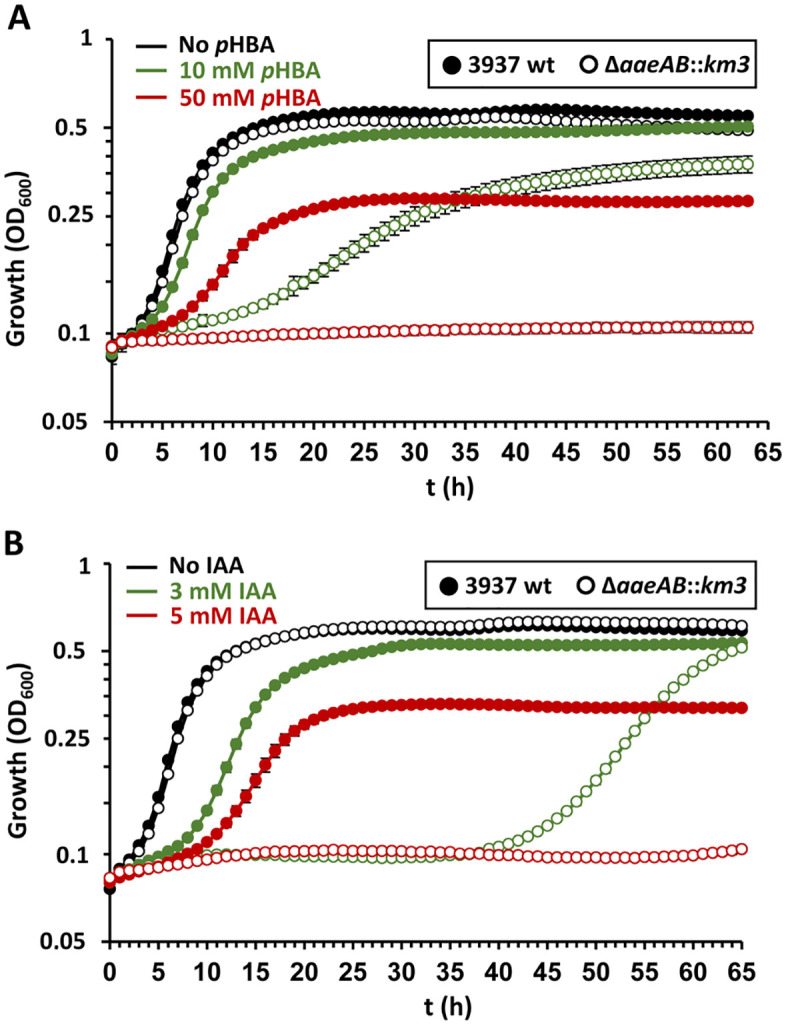
Effect of different concentrations of *p*-hydroxybenzoate (*p*HBA) and IAA on the growth kinetics of *D. dadantii* 3937 strains. Shown is the growth of *D. dadantii* strains in minimal medium in the absence or presence of different concentrations of *p*HBA (A) and IAA (B). The bioassays were repeated at least three times and representative results of one biological replicate are shown. Data are means and standard deviations of five technical replicates. Some standard deviations are minor and are not visible in the corresponding growth curves. Growth was measured using Bioscreen Microbiological Growth Analyser (Oy Growth Curves Ab Ltd, Helsinki, Finland).

The corresponding AaeXAB components in *E. coli* and *D. dadantii* 3937 share 57.7-70.8% sequence identity, supporting orthologous efflux pump-encoding genes. To investigate the functional role of the AaeXAB pump in *D. dadantii* 3937, we compared the growth of the wild-type and ∆*aaeAB* strains in the presence of IAA and different aromatic compounds. The ∆*aaeAB* mutant grew similarly to the wild-type strain under standard conditions but exhibited increased sensitivity to *p*-hydroxybenzoate and salicylate, as indicated by an extended lag phase, slower growth rate, and reduced final cell density, whereas its growth remained unaffected by other aromatic compounds like benzoate (Figs 4A and Fig C in S1 Text). Consistently, the minimum inhibitory concentrations (MICs) for *p*-hydroxybenzoate and salicylate were approximately twofold lower in the ∆*aaeAB* mutant than in the wild-type (Table A in S1 Text). Notably, the ∆*aaeAB* mutant was markedly more sensitive to IAA, exhibiting a MIC value approximately fourfold lower than that of the wild-type strain (Table A in S1 Text), and showed a pronounced lag phase beginning at 1 mM IAA (Figs 4B and Fig D in S1 Text), highlighting the importance of the AaeXAB pump in protecting *D. dadantii* 3937 from auxin-induced stress.

The *aaeXAB* gene cluster in *D. dadantii* 3937 has an upstream, divergently transcribed gene encoding the LysR-type transcriptional regulator AaeR. In *E. coli*, AaeR regulates *aaeXAB* expression [51], although its ligand specificity remains unresolved. To investigate the regulatory mechanisms governing AaeXAB expression, we overexpressed and purified the ligand binding domain (LBD) of AaeR (AaeR-LBD) and assessed its ability to bind IAA and *p*-hydroxybenzoate using isothermal titration calorimetry (ITC). These assays yielded only small, uniform heat changes (Fig EA in S1 Text), indicative of a lack of interaction with AaeR-LBD. To rule out ligand recognition outside the LBD, ITC assays were repeated with the full-length AaeR, which also showed no evidence of binding (Fig EB in S1 Text). Finally, a high-throughput differential scanning fluorimetry screen [52] was conducted using ~450 compounds from the Biolog Phenotype MicroArray plates PM1, PM2A, PM3B, PM4A, and PM5, covering diverse carbon, nitrogen, sulfur, or phosphorus sources (Fig F in S1 Text). None of the tested compounds thermally stabilized AaeR-LBD (Fig G in S1 Text), indicating no detectable interaction with the screened potential ligands. To investigate the role of AaeR in IAA efflux, we generated an *aaeR* mutant and measured IAA levels in culture supernatants by GC-MS, which were similar to those in the wild-type strain (Fig 1), supporting that AaeR does not play a role in IAA efflux.

IAA, *p*-hydroxybenzoate, and salicylate are plant-derived compounds present in plant exudates [53,54]. To assess the role of the AaeXAB efflux pump in the virulence of *D. dadantii* 3937, we performed infection assays in potato tubers and plants. Whereas the wild-type and the ∆*aaeAB* mutant exhibited comparable virulence in potato tubers (Fig H in S1 Text), the ∆*aaeAB* mutant exhibited a marked tendency toward reduced bacterial populations (~35% reduction) in potato leaves compared with the parental strain (Fig I in S1 Text). This observation likely reflects the greater chemical complexity of foliar tissues relative to potato tubers, which consist mainly of starch [55]. No differences were observed between the wild-type and the ∆*aaeAB* mutant in growth media simulating growth conditions encountered in leaves and potato tubers (Fig J in S1 Text), nor in the production of siderophores or plant cell wall–degrading enzymes (PCWDEs) (Fig K in S1 Text), which represent major virulence determinants of *D. dadantii* 3937 [45]. These results suggest that the absence of the AaeAB efflux pump may impair the detoxification of specific leaf-derived compounds with inhibitory activity.

### The AaeXAB efflux pump is widely conserved among plant-associated Pseudomonadota

**Fig 5:**
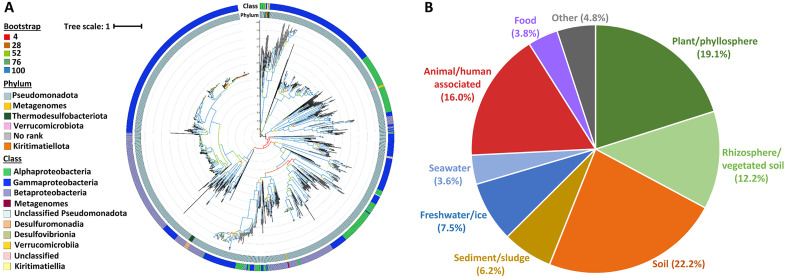
Phylogenetic distribution and isolation sources of bacterial strains carrying the *aaeXAB* locus. A, Maximum-likelihood (ML) phylogenetic tree of 774 taxa possessing the *aaeXAB* genes. Each leaf represents a concatenated AaeX-A-B sequence triplet per organism (n = 774). The concentric scale in the circular representation indicates branch lengths in expected amino acid substitutions per site as estimated under the LG + G4 + F model. Branch support values are shown using colored branches. Inner and outer rings indicate the taxonomic distribution of *aaeXAB* clusters across bacterial phyla and classes, respectively. B, Isolation sources of bacterial strains encoding the AaeXAB efflux pump shown in (A). Information on isolation sources was obtained from NCBI BioSample, the Integrated Microbial Genomes & Microbiomes (IMG/M) platform of the Joint Genome Institute, and through literature searches. Complete details of the bacterial strains used to generate this figure are provided in S1 Table.

Given the role of the AaeXAB efflux pump in conferring resistance to diverse plant-derived compounds (Figs 4 and Fig C in S1 Text) and its contribution to plant virulence (Fig I in S1 Text), we hypothesized that this pump may be commonly present in plant-associated bacteria. To test this hypothesis, we first analyzed the phylogenetic distribution of the *aaeXAB* genes by examining annotated genomes available in the NCBI database. Our analysis identified *aaeXAB*-containing clusters in at least 774 bacterial taxa, with their occurrence largely restricted to the phylum Pseudomonadota (98.3%) (Fig 5 and S1 Table). Although the genetic organization of these clusters varied, 378 (~48.8%) contained associated genes encoding LysR-type transcriptional regulators (S1 Table). The phylogenetic reconstruction revealed well-supported, independent radiations, within each major Pseudomonadota lineage, suggesting that this efflux system has undergone lineage-specific diversification associated with environmental adaptation. At the genus level, the *aaeXAB* cluster was most frequently detected in *Pseudomonas* (23.5%), but was also abundant in other genera such as *Paraburkholderia* (5.7%), *Burkholderia* (4.8%), *Vibrio* (2.8%), *Caballeronia* (2.5%), and *Bradyrhizobium* (1.4%) (Fig 5A and S1 Table). Notably, examination of the isolation sources revealed that bacteria carrying the *aaeXAB* cluster were predominantly recovered from plants and plant-associated environments (Fig 5B and S1 Table). In addition, isolates harboring the *aaeXAB* cluster were also isolated from soil and freshwater environments (Fig 5B and S1 Table) - two habitats typically enriched in bacteria that interact with plants. Together, these findings support a conserved role for the AaeXAB efflux pump in promoting bacterial fitness and persistence within plant-associated niches.

### The MarR-type transcriptional regulator DDA3937_RS07305 binds indole

**Fig 6:**
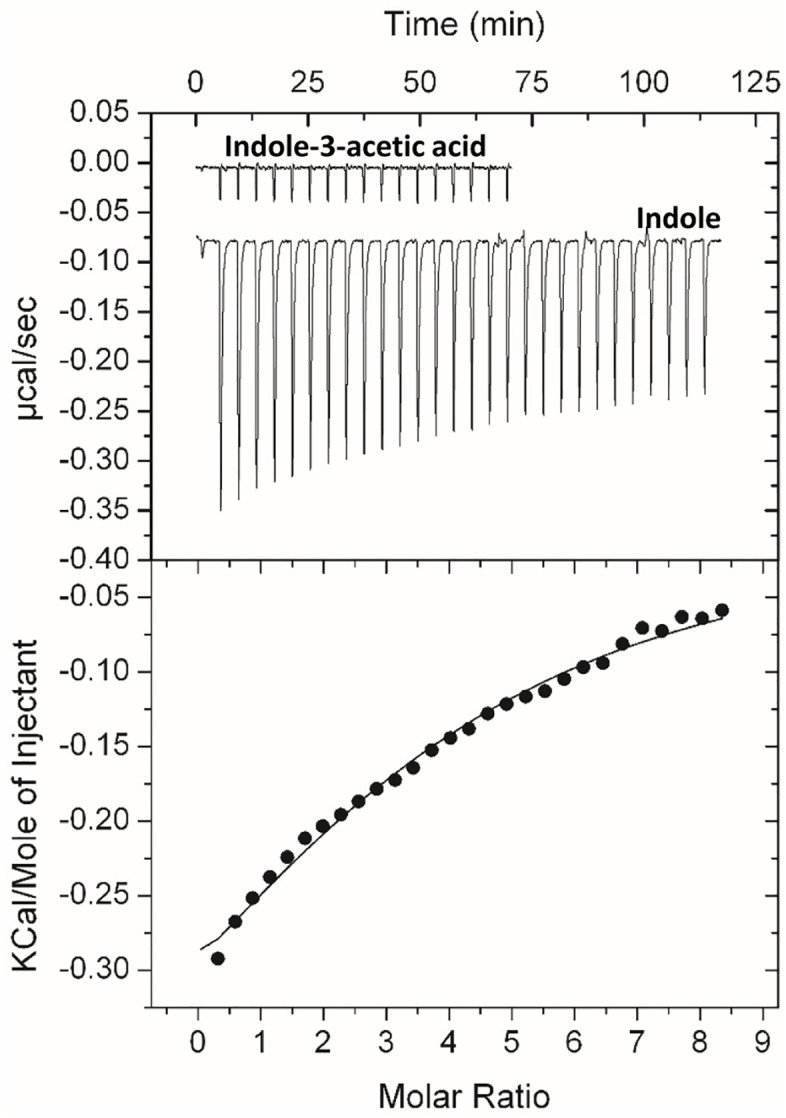
The MarR-type transcriptional regulator DDA3937_RS07305 binds indole. Shown are isothermal titration calorimetry analysis of DDA3937_RS07305 with indole and IAA. Upper panel: Raw data for the titration of 40 μM of DDA3937_RS07305 with 9.6 μL aliquots of 2-5 mM indole and IAA solutions. Lower panel: concentration-normalized and dilution heat–corrected integrated raw data. The line represents the best fit using the one binding site model of the MicroCal version of ORIGIN. The derived thermodynamic parameters for indole binding were: *K*_D_ = 180 ± 26 µM; ∆H = -0.6 ± 0.06 kcal/mol.

Our transcriptomic analysis revealed that the gene *DDA3937_RS07305*, encoding an uncharacterized MarR-type transcriptional regulator, was downregulated in the wild-type strain (Table 1). In a previous transcriptomic study, the *DDA3937_RS07305* gene was shown to exhibit modest, opposite changes in expression in response to environmental stimuli relevant to plant infection [56]. MarR-family proteins typically function as transcriptional repressors [57,58], with their regulatory activities modulated by ligand binding, including plant-derived compounds [57–59]. Among these ligands, several MarR-type regulators have been reported to bind IAA [35,60]. To identify potential ligands for DDA3937_RS07305, the protein was purified and subjected to high-throughput differential scanning fluorimetry-based assays using the library of ~450 compounds from Biolog described above. This screen did not reveal any candidate ligands. Subsequently, we performed ITC assays with IAA and its biosynthetic precursors, L-Trp and indole. While no binding was detected for IAA or L-Trp, DDA3937_RS07305 bound indole with a dissociation constant (*K*_D_) of 180 ± 26 µM (Fig 6), suggesting that it may function as an indole-responsive regulator. The three-dimensional structures of several MarR-type regulators in complex with IAA have been determined, allowing the identification of key residues involved in IAA recognition [35]. These residues are not conserved in DDA3937_RS07305 (Fig L in S1 Text), indicating that indole recognition by this regulator involves alternative residues.

To investigate the role of DDA3937_RS07305 in IAA biosynthesis in *D. dadantii* 3937, we generated a mutant defective in this regulator and quantified IAA levels in culture supernatants using GC–MS. The results showed that the *DDA3937_RS07305* mutant exhibited IAA levels comparable to those of the wild-type strain (Fig 1), indicating that this indole-responsive regulator does not modulate auxin biosynthesis.

### Indole-3-acetic acid biosynthesis contributes to the *in* *planta* virulence and competitive fitness of *D. dadantii*

**Fig 7:**
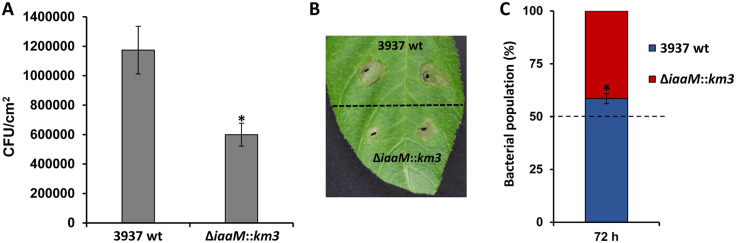
Role of IAA synthesis in the virulence and competitive colonization fitness of *D. dadantii* 3937 in potato plants. A, Virulence of *D. dadantii* 3937 strains in potato leaves, expressed in CFU/cm^2^. Virulence was assessed based on bacterial population sizes in potato leaves at 72 h after syringe-infiltration (5 x 10^7^ CFU/mL). Data were corrected based on the initial inoculum and are means and standard errors from three independent experiments conducted in triplicate. *P  <  0.05, Kruskal-Wallis test. B, Representative image of symptoms observed 72 h after inoculation in the virulence assays shown in panel (A). C, Competitive colonization fitness of *D. dadantii* strains. Shown are the proportions of CFU recovered 72 h after co-inoculation in potato leaves. Data were corrected based on the initial inoculum and are means and standard errors from three independent experiments conducted in triplicate. *P  <  0.01, Kruskal-Wallis test.

Given the role of IAA in the virulence of several bacterial phytopathogens [5,15] and the *in planta* induction of *iaaM* in *D. dadantii* 3937 [48], we investigated the contribution of IAA biosynthesis to the virulence of *D. dadantii* 3937 in potato plants. The *iaaM* mutant exhibited a significant reduction in population size compared with the wild-type, reaching approximately half the density of the parental strain (Fig 7A, 7B). We then assessed the impact of IAA production in colonization fitness in potato leaves using competition assays. The results revealed that the *iaaM* mutant displayed a modest but statistically significant reduction in competitive fitness relative to wild-type (Fig 7C). To investigate whether these phenotypes were associated with alterations in the production of major virulence determinants of *D. dadantii* 3937 [45], we evaluated siderophore and PCWDEs production in both the wild-type and the *iaaM* mutant. Consistent with the transcriptomic data (Table 1), the *iaaM* mutant displayed wild-type levels of cellulolytic, pectinolytic, and proteolytic activities, among others, as well as unchanged siderophore production (Fig M in S1 Text). Collectively, these results indicate that IAA biosynthesis contributes to the virulence and competitive fitness of *D. dadantii* 3937 during plant infection, independently of PCWDEs and siderophore production.

### Distinct regulators control IAA biosynthesis in *D. dadantii*

To our knowledge, no regulators of IAA biosynthesis have been identified in *Dickeya* species to date. To address this gap, we generated a series of *D. dadantii* 3937 mutants defective in candidate regulatory genes and quantified their IAA production levels by GC-MS. Because IAA biosynthesis in *D. dadantii* 3937 peaks during stationary phase [49] and quorum-sensing has been implicated in auxin synthesis in other bacteria [36,61,62], we investigated the role of the two quorum-sensing systems of *D. dadantii* 3937 [63–65]. The ExpIR system relies on N-acyl-homoserine lactone (AHL) signaling, whereas the *Dickeya*-specific Vfm (virulence factor-modulating) system depends on a still-uncharacterized signaling molecule. The *expI* mutant, lacking the AHL synthase, produced ~37% more IAA than the wild-type (Fig 1). In contrast, a *vfmI* mutant - defective in the gene encoding the histidine kinase putatively responsible for signal perception and activation of the Vfm regulatory cascade [63–65] - exhibited an approximately 75% reduction in IAA levels (Fig 1). This reduction persisted in the expI vfmI double mutant, which produced IAA levels comparable to those of the *vfmI* single mutant (Fig 1). Given that L-tryptophan is the main precursor of IAA [15], we next examined the contributions of TrpR and TyrR - two key transcriptional regulators of aromatic acid metabolism and transport [40,66,67]. We first focused on TyrR, a regulator shown to bind aromatic amino acids (e.g., L- Tyr, L-Trp and L-Phe) in *E. coli* [68] and *S. plymuthica* [36], and previously reported to positively regulate IAA synthesis in several bacterial species [36,69]. The *tyrR* mutant exhibited a ~64% reduction in IAA levels (Fig 1). Similarly, a mutant defective in the tryptophan repressor TrpR, which has been shown to bind L-tryptophan and IAA in other species [25,70], displayed a significant ~33% decrease in IAA relative to the wild-type (Fig 1). Finally, we investigated the role of the LysR-type transcriptional regulator LrhA (homologue of PecT and HexA) - a global regulator that modulates processes relevant to plant interaction, including motility, secondary metabolism, and the production of plant cell wall-degrading enzymes [71–75]. Disruption of *lrhA* caused a striking 4.75-fold increase in IAA production (Fig 1) - a phenotype that was partially complemented by *in trans* expression of *lrhA* from a pBBR-based plasmid (Fig A in S1 Text).

**Fig 8:**
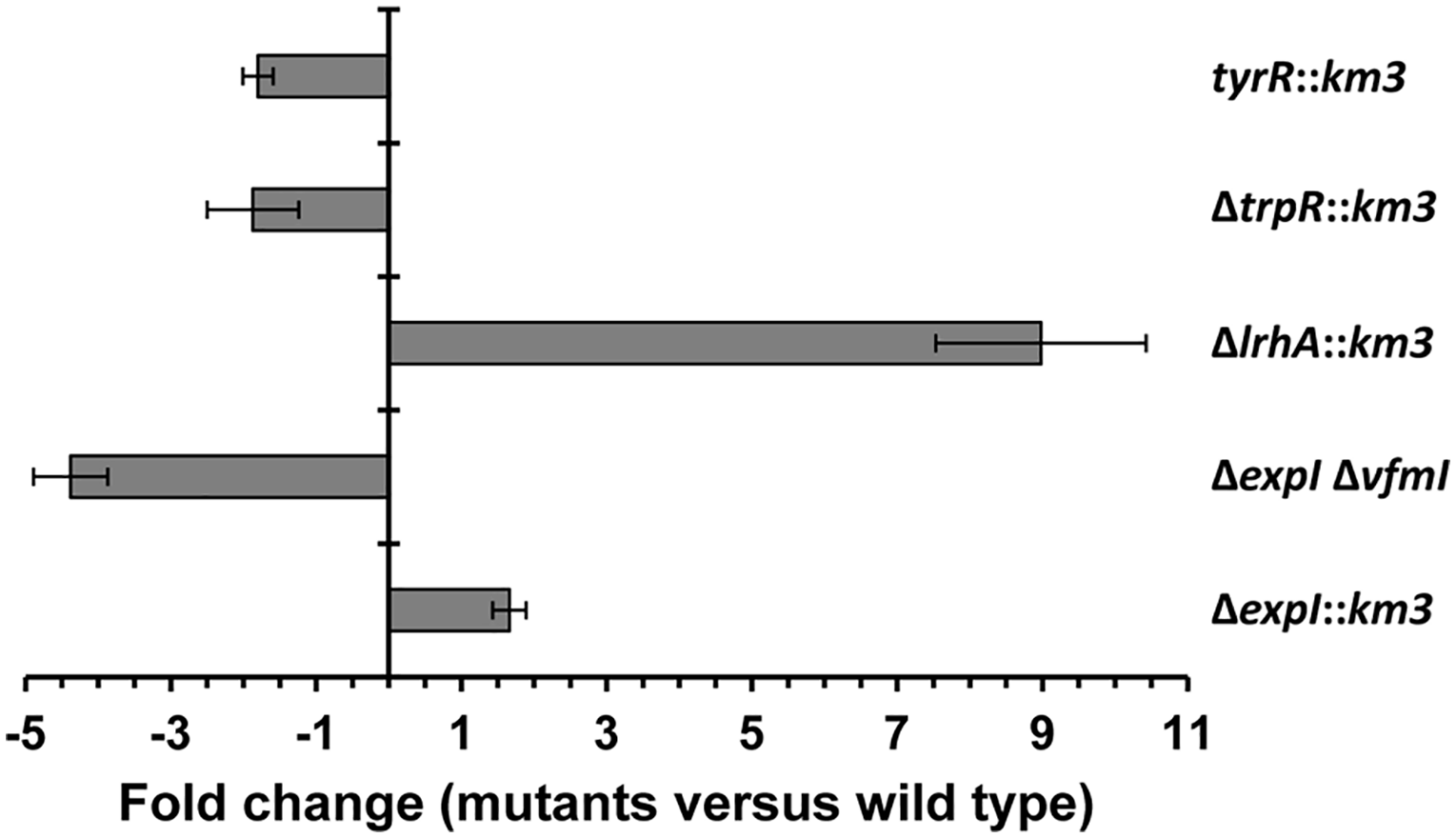
Impact of quorum-sensing and different transcriptional regulators on the expression of the IAA biosynthetic gene *iaaM.* Shown are the fold changes of *iaaM* mRNA levels in *D. dadantii* 3937 mutants relative to the wild-type strain, as determined by RT-qPCR under the same conditions used for the RNA-seq analysis. Data represent the means and standard deviations of three biological replicates, each performed in triplicate. RT–qPCR analysis of the *vfmI* mutant strain did not yield detectable amplification of *iaaM* transcripts using different primer pairs, preventing the determination of fold-change values but suggesting a markedly reduced expression of the *iaaM* gene in this mutant background.

To investigate whether altered IAA production in these mutants correlated with changes in the expression of IAA biosynthetic genes, we quantified *iaaM* transcript levels by RT-qPCR. The results revealed a strong correlation between *iaaM* transcript abundance in the mutants (Fig 8) and their corresponding IAA production levels (Fig 1). Collectively, these results indicate the existence of an intricate regulatory network modulating IAA biosynthesis in *D. dadantii* 3937, with multiple regulatory systems exerting either positive or negative control over auxin production.

## Discussion

Inter-kingdom communication between plants and their associated microbiota is highly dynamic and mediated by a diverse repertoire of signaling molecules [54,59,76–80]. This communication is not only crucial for plant health and growth, but also plays an important role in modulating the physiology, metabolism, and virulence programs of the plant microbiome, as well as its composition and structure [54, 76–82]. Among these signaling molecules, a growing body of experimental evidence highlights the central bidirectional role of IAA in plant-microbe interactions [5,15,16,25], as this auxin is synthesized by both plants and their associated microbes. Specifically, IAA regulates a broad spectrum of bacterial processes during plant–microbe interactions [5,15,25,27,28,34], including, as demonstrated in this study, the modulation of resistance to stresses prevalent in plant-associated niches [23,25,27,29]. Moreover, IAA can serve as a nutrient for PAB in an auxin-regulated process [35,37,60,83]. Collectively, these findings support its role as a regulatory signal in plant–bacterium interactions, further reinforced by evidence that variations in IAA concentration within root exudates influence the composition of the rhizospheric microbiota [84].

Here, we demonstrate that IAA biosynthesis plays an important role for the virulence of *D. dadantii*. This global phytopathogen relies on several major virulence determinants, including PCWDEs production, chemotaxis and motility, secretion systems, siderophore and antioxidant compound biosynthesis, as well as extensive metabolic versatility [44,45,80,85,86]. Previous studies provided evidence that IAA production by D. dadantii may modulate pectate lyase production and type III secretion system expression [47]. However, our transcriptomic and phenotypic analyses did not reveal any role for IAA synthesis in these processes under the tested conditions. Therefore, we hypothesize that IAA production by *D. dadantii* contributes to virulence by interfering with plant auxin signaling through diverse, non-exclusive mechanisms. First, through cross-talk with other signaling pathways that regulate plant defense responses. Indeed, experimental evidence indicates that IAA antagonizes salicylic acid–mediated defense signaling [5,87–89] and interacts with other hormone signaling networks involved in plant defense, such as the jasmonic acid pathway [5,89,90]. Second, IAA production during infection may promote plant susceptibility by disrupting host auxin homeostasis, thereby stimulating plant cell division and elongation, inducing cell wall loosening [91,92], and ultimately facilitating cell wall degradation by phytopathogenic PCWDEs.

During plant interactions, PAB must fine-tune their metabolism, physiology, and gene expression to cope with the diverse stresses encountered in plant-associated niches. These adaptive mechanisms include biofilm formation [93–95], production of osmoprotectants [45,56,96], synthesis of antioxidant enzymes [56,97–99], and expression of detoxification systems that counteract toxic plant-derived compounds [99–102], among others. The AaeXAB efflux pump was initially identified in *E. coli* as being involved in resistance to *p*-hydroxybenzoic acid [51] and we subsequently demonstrate that its expression is induced in the rhizosphere biocontrol agent *Serratia plymuthica* in the presence of exogenous IAA [25], but not in response to endogenous IAA [36], where it confers resistance to this auxin and to *p*-hydroxybenzoic acid [25]. Here, we investigate, for the first time, the AaeXAB efflux pump in a bacterial phytopathogen and demonstrate that in *D. dadantii* it is induced by both endogenous and exogenous IAA, playing a pivotal role in the resistance to plant-derived compounds and defense-related signaling molecules, including IAA and salicylic acid. IAA in plant environments originates not only from plants but also from plant-associated microorganisms, which commonly produce substantial amounts of this auxin [13,15,17,32]. While micromolar concentrations of IAA have been reported in the rhizosphere [103,104], local concentrations may be higher in specific plant niches, as supported by several observations: (i) IAA catabolism enhances bacterial fitness in the rhizosphere [37]; (ii) IAA in root exudates influences microbial community composition [84]; and (iii) bacterial IAA catabolism can mitigate plant growth inhibition caused by root-associated microbial communities [20]. No change in *aaeXAB* expression was previously detected in response to common stresses typically encountered during bacterial adaptation to plant environments, such as low pH, oxidative, or osmotic stress [56], supporting that AaeXAB regulation in *D. dadantii* is specifically linked to plant-derived signals. To the best of our knowledge, MatE [105] and AEC [106] remain the only bacterial transporters known to date to be involved in IAA secretion, making AaeXAB the first IAA efflux pump described in the *Dickeya* genus. We also linked the AaeXAB pump to *D. dadantii* virulence *in planta* and, given its down-regulation in the *iaaM* mutant, this pump may also contribute to the reduced virulence of this strain. Several efflux pumps in *D. dadantii* have been associated with plant infection [102], further emphasizing their importance as key virulence determinants. Given its role in auxin secretion, AaeXAB likely functions as an important component of IAA signaling among PAB and contributes to the inter-kingdom chemical dialogue between plants and their associated microbiota.

Several plants and bacteria produce indole from L-tryptophan via indole-3-glycerol phosphate lyases and tryptophanases, respectively [107–109]. Similar to IAA, indole functions as an interspecies, intraspecies, and inter-kingdom signal that controls diverse processes relevant for bacteria-host interactions, including plant growth and development, bacterial motility and chemotaxis, biofilm formation, stress tolerance, antibiotic resistance, and virulence [107–113]. We showed here that endogenous IAA modulates the expression of an indole-response regulator, suggesting cross-talk between IAA- and indole-mediated regulatory networks. Additionally, the IAA-dependent regulation of a salicylic acid and *p*-hydroxybenzoic acid efflux pump supports the existence of cross-regulation between auxins and aromatic plant defense compounds. In this context, bacterial-derived indole has been shown to interfere with auxin signaling in plants [114]. Collectively, these findings illustrate the complexity of chemical communication in plant-bacteria interactions. Future research will aim to unravel the interplay between IAA- and indole-mediated signaling in PAB.

## Conclusions

**Fig 9:**
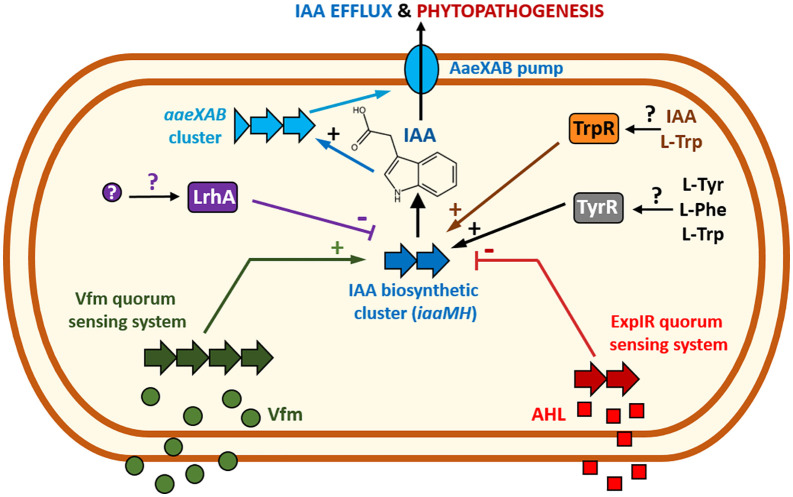
Model of the regulatory network controlling IAA biosynthesis and efflux in *D. dadantii.* The Vfm quorum-sensing system and the *N*-acyl homoserine lactone (AHL)-mediated quorum-sensing system ExpIR act as activator and repressor, respectively, of IAA production in *D. dadantii* 3937. Likewise, the transcriptional regulators TyrR and TrpR positively regulate IAA biosynthesis, whereas LrhA acts as a strong repressor. TyrR and TrpR have been shown in the enterobacterial species *E. coli* and *S. plymuthica* to bind L-Trp, L-Phe, and L-Tyr, and L-Trp and IAA, respectively [25,36,68,70]. Given the high sequence identity (61.1% - 73.1%) between these regulators and their homologs in *D. dadantii*, they are likely to recognize the same ligands in this species. IAA functions as a bacterial signaling molecule that induces the expression of the AaeXAB efflux pump, which exports IAA to contribute to the regulation of virulence and plant adaptation in *D. dadantii*.

IAA functions as a central inter-kingdom signal in plant–microbe interactions and is increasingly recognized as a bacterial signaling molecule that modulates diverse processes critical for plant host adaptation. Yet, the mechanisms by which IAA exerts its regulatory activities and the control of its production in bacteria remain poorly understood. Here, we demonstrate that IAA biosynthesis in *D. dadantii* is pivotal for virulence, colonization fitness, and adaptation to plant environments. Specifically, IAA regulates the expression of the AaeXAB efflux pump, which confers resistance to plant defense compounds, thereby promoting successful plant infection and colonization. These findings identify a new regulatory connection between IAA signaling, bacterial resistance mechanisms, and plant pathogenicity, opening avenues for the development of efflux pump inhibitors as anti-virulence strategies against this phytopathogen [115–117]. Considering the importance of IAA in *D. dadantii* pathogenicity, we show that bacterial auxin synthesis is tightly regulated through the coordinated action of multiple transcriptional regulators and quorum-sensing systems (Fig 9). Furthermore, our data support cross-talk between IAA- and indole-mediated signaling pathways, highlighting the complexity of chemical communication networks that govern bacterial adaptation and intra- and inter-kingdom interactions within the plant microbiome. Given the widespread distribution of IAA biosynthesis across the Tree of Life [12,15] and the diversity of biological processes it regulates [15,118–121], auxins have emerged as global signals in life. Beyond their ecological significance, they hold enormous potential for diverse clinical and biotechnological applications, including the biosynthesis of medically and industrially relevant compounds, microbiome engineering, and the development of anti-virulence strategies, among others [121–124]. Future studies dissecting the molecular mechanisms underlying IAA-mediated regulation and its interplay with other microbial signals will be essential for advancing our understanding of microbial behavior, inter-kingdom communication, and the ecological dynamics of the plant microbiome.

## Materials and methods

### Bacterial strains, plasmids, oligonucleotides, and culture conditions

Bacteria and plasmids used in this study are described in Table B in S1 Text, whereas oligonucleotides are listed in Table C in S1 Text. *Dickeya dadantii* strains were grown routinely at 28 °C, unless otherwise indicated, in lysogeny broth (LB) or minimal medium (0.1% (w/vol) (NH_4_)_2_SO_4_, 0.41 mM MgSO_4_, 40 mM K_2_HPO_4_, 14.7 mM KH_2_PO_4_, pH 6.9–7.1) with 15 mM glucose as carbon source, unless otherwise indicated. *Escherichia coli* strains were grown at 37 °C in LB. *E. coli* DH5α was used as a host for gene cloning. Media for propagation of *E. coli* β2163 were supplemented with 300 μM 2,6-diaminopimelic acid. When appropriate, antibiotics were used at the following final concentrations (in μg/mL): ampicillin, 100; kanamycin, 50; streptomycin, 50.

### *In vitro* nucleic acid techniques

Plasmid DNA was isolated using the NZY-Miniprep kit (NZY-Tech). For DNA digestion, alkaline phosphatase and ligation reactions, manufacturers’ instructions were followed (New England Biolabs and Roche). DNA fragments were recovered from agarose gels using the Qiagen gel extraction kit. PCRs were purified using the Qiagen PCR clean-up kit. The Mix & Go transformation kit (Zymo Research, Cat. No.: T3002) was used to prepare *E. coli* competent cells, and transformations were performed using standard protocols [125]. Phusion high-fidelity DNA polymerase (Thermo Fisher Scientific) was used for the amplification of PCR fragments.

### Marker exchange mutagenesis and construction of complementation plasmids

Mutant strains defective in *iaaM* (*DDA3937_RS01905*), *aaeAB* (*DDA3937_RS01475/DDA3937_RS01480*), *aaeR* (*DDA3937_RS01465*), *DDA3937_RS07305*, *expI* (*DDA3937_RS20725*), *vfmI* (*DDA3937_RS20815*), *trpR* (*DDA3937_RS18450*), *tyrR* (*DDA3937_RS12390*) and *lrhA* (*DDA3937_RS14340*) were constructed by homologous recombination using derivative plasmids of the suicide vector pKNG101, namely plasmids pMAMV394, pMAMV468, pKNG-AaeR, pKNG-MarR_7305, pMAMV438, pMAMV233, pKNG-TrpR, pKNG-TyrR, and pKNG-LrhA, respectively (Table B in S1 Text). The double *expI*/*vfmI* mutant was generated using pMAMV233 and the ∆*expI*::*km3* strain as recipient. The plasmids were transferred to *D. dadantii* strains by biparental conjugation using *E. coli* β2163. Cells were then spread onto LB plates containing 50 μg/mL streptomycin. Merodiploid colonies were selected and further spread onto LB plates containing 10% (w/vol) sucrose to isolate derivatives that had undergone a second cross-over event during marker exchange mutagenesis. All plasmid constructs and the resulting mutant strains were verified by PCR and sequencing carried out at Stab Vida (Caparica, Portugal), using oligonucleotides listed in Table C in S1 Text.

To generate plasmids for complementation assays, the *iaaM* and *lrhA* genes were amplified using primers listed in Table C in S1 Text and cloned into pBBR1MCS-5_START. The resulting plasmids were transformed into *D. dadantii* 3937 strains by electroporation. Complementation plasmids were verified by PCR and sequencing using oligonucleotides listed in Table C in S1 Text.

### Growth experiments

*Dickeya dadantii* strains were grown overnight in M9 minimal medium containing 15 mM glucose, M63 minimal medium (2.0 g/L (NH₄)₂SO₄, 13.6 g/L KH₂PO₄, 0.5 mg/L FeSO₄·7H₂O, 0.2% (vol/vol) glycerol, 1 mM MgSO₄, 1 µg/mL thiamine) supplemented with 0.2% (w/vol) sucrose and 0.2% (w/vol) polygalacturonic acid, or potato dextrose broth (26.5 g/L; Condalab, Spain). The cultures were washed twice with M9 salts and diluted to an OD_600_ of 0.02 in M9 minimal medium containing 15 mM glucose supplemented with different concentrations of *p*-hydroxybenzoate, indole-3-acetic acid, salicylate or benzoate, all prepared in the same buffered M9 minimal medium and adjusted to pH 7.0. Two-hundred microliters of these cultures were transferred to microwell plates and growth (OD_600_) was monitored at 28 ºC using a Bioscreen Microbiological Growth Analyser (Oy Growth Curves Ab, Helsinki, Finland).

### RNA extraction, cDNA synthesis and quantitative real-time PCR analyses

Total RNA was extracted by using TRI Reagent (Invitrogen) followed by Turbo DNase treatment (Ambion) and RNA clean-up with RNeasy Mini Kit (Qiagen), according to manufacturers´ instructions. The RNA concentration was determined spectrophotometrically and RNA degradation and contamination was assessed by electrophoresis on 2% (w/vol) agarose gels. The synthesis of cDNA was performed using random hexamers (GE Healthcare) and SuperScript II reverse transcriptase (Invitrogen) in a 25 µL reaction volume with 1 µg of total RNA and incubation at 42 ºC for 2 h. RT-qPCRs were performed as described previously [126] using primers described in supplementary Table C in S1 Text. RT-qPCR amplifications were performed using the iQ SYBR Green supermix (Bio-Rad) in a MyiQ2 Two-Color Real-Time PCR Detection System (Bio-Rad) associated with iQ5 optical system software (version 2.1.97.1001). To confirm the absence of contaminating genomic DNA, control PCRs were carried out using samples without reverse transcriptase as templates. Melting curve analyses were conducted to ensure the amplification of a single product. The relative gene expression was calculated using the critical threshold (ΔΔCt) method [127] using the *gyrB* gene as reference for data normalization.

### RNA sequencing

RNA sequencing was performed at Novogene Company Limited (United Kingdom) using samples from the wild-type *D. dadantii* 3937 and the ∆*iaaM*::*km3* mutant grown in minimal medium supplemented with 0.25 mg/mL L-tryptophan (IAA precursor) and harvested at the early stationary phase of growth (OD_600_ ~ 1.4). Prior to shipping on dry ice, RNA integrity and quantification were assessed using the RNA Nano 6000 Assay Kit of the Bioanalyzer 2100 system (Agilent Technologies, CA, USA). Upon receipt, Novogene assessed RNA purity using the NanoPhotometer spectrophotometer (IMPLEN, California, USA) and further verified RNA integrity and concentration with the Agilent 5400 system (Agilent Technologies, California, USA). One microgram of total RNA per sample (three biological replicates per strain) was used to construct the sequencing libraries. These libraries were generated by using NEBNext Ultra Directional RNA Library Prep Kit for Illumina (New England Biolabs; #E7530), following the manufacturer's recommendations. Prior to library construction, ribosomal RNA was depleted with Illumina Ribo-Zero Plus rRNA Depletion Kit (Illumina; Ref. 20037135), according to the manufacturer's instructions. The resulting libraries were quantified by quantitative PCR and the inserts were measured using a LabChip GX instrument, using the LabChip NGS 3K reagent kit (PerkinElmer; CLS960013). Libraries were sequenced using the NovaSeq 6000 Illumina platform and paired-end reads were generated.

### RNA-Seq data processing and differential expression analysis

Raw Illumina RNA-Seq reads were first subjected to quality control and adapter trimming using Fastp v0.23.2 [128]. This step included removal of adapter sequences, filtering of low-quality reads (Phred score < 30), and exclusion of reads shorter than 50 bp. The quality of both raw and processed reads was then assessed with FastQC [129] to ensure the absence of adapter contamination and to evaluate per-base sequence quality. High-quality reads were aligned to the reference genome (*D. dadantii* 3937, RefSeq ID = GCF_000147055) using STAR v2.7.11b [130] with default parameters. The resulting alignment files (sorted BAM format) were used as input for transcript assembly and quantification with StringTie2 [131], which generated both transcript-level and gene-level expression estimates in the form of read count matrices. Differential expression analysis was carried out in Python using pyDESeq2 [132], an implementation of the DESeq2 statistical model [133]. Count matrices and sample metadata were loaded into Spyder v6.1.0 (Python 3.13 environment), and the analysis was performed following standard DESeq2 procedures: library size normalization, dispersion estimation, model fitting, and Wald tests for pairwise contrasts between experimental conditions. Genes with an adjusted *p*-value (Benjamini–Hochberg false discovery rate, FDR) ≤ 0.05 and an absolute log₂ fold change ≥ 0.5 were considered significantly differentially expressed. Visualization of differential expression results was performed using matplotlib, seaborn, and scikit-learn packages. Gene functional annotation and pathway enrichment analyses were conducted by mapping protein-coding sequences to KEGG Orthology (KO) terms via the KEGG REST API, followed by over-representation analysis (ORA) and gene set enrichment analysis (GSEA) using gseapy [134,135].

### Phylogenetic analyses

AaeXAB protein sequences were retrieved from UniProt filtering by gene name, protein product and protein domain architectures. Each gene was independently aligned using MAFFT v7.520 [136] with default parameters. The resulting alignments were inspected to ensure homology and subsequently processed to identify orthologous copies belonging to the same organism and operon. The best-fit evolutionary model for each alignment was determined using ModelTest-NG v0.1.7 [137], which identified LG + G4 + F as the optimal model under the Bayesian Information Criterion (BIC) for all three genes. A custom Python script was developed to parse the UniProt metadata table and matched entries across the three protein sequences based on the Organism and locus_tag identifiers, to select the most likely operonic triplet (AaeX, AaeA, AaeB) according to their ORF proximity, penalizing high gap fractions and deviations from gene-specific median sequence lengths. The three corresponding aligned sequences were then concatenated into a single alignment per organism, producing a concatenated AaeX–AaeA–AaeB supermatrix that included 774 taxa. A partitioned maximum-likelihood (ML) phylogenetic tree was reconstructed using RAxML-NG v1.2.2 [138], assigning the LG + G4 + F substitution model to each protein as a separated partition. Branch support was evaluated using 100 bootstrap replicates. The resulting best-scoring ML tree was rooted at the midpoint and plotted using the Interactive Tree of Life (*iTOL*) v7.2.2 [139]. The identification of regulators associated with AaeXAB systems was performed as described in the supplementary materials and methods.

### Protein overexpression and purification

*E. coli* BL21(DE3) harboring plasmids pET28b-AaeR_3937, pET28b-AaeR-LBD_3937, and pET28b-DDA3937_RS07305 were grown in 2 L Erlenmeyer flasks containing 500 mL LB medium supplemented with kanamycin. The cultures were grown at 30 ºC under continuous stirring (200 rpm). When the cultures reached an OD_600_ of 0.5, protein expression was induced by adding 0.25 mM isopropyl-β-d-1-thiogalactopyranoside (IPTG) and growth was continued overnight at 18 ºC. Cells were then harvested by centrifugation at 10,000 x *g* for 20 min at 4 ºC. Proteins were purified by metal affinity chromatography. Briefly, the cell pellets of AaeR, AaeR-LBD and DDA3937_RS07305 were resuspended in buffer A (30 mM Tris, 300 mM NaCl, 10% (vol/vol) glycerol, 10 mM imidazole, 5 mM β-mercaptoethanol; pH 8.5) containing 1 mM phenylmethylsulfonyl fluoride (PMSF) protease inhibitor (Thermo Fisher Scientific) and benzonase (Merck). Cells were lysed using a French press at a gauge pressure of 62.5 lb/in^2^. After centrifugation at 10,000 x *g* for 1 h, the supernatant was loaded onto a 5 mL HisTrap column (Amersham Bioscience) pre-equilibrated with buffer A. Proteins were eluted with a gradient of 40–500 mM imidazole in the same buffer. Protein-containing fractions were pooled and dialyzed into 5 mM Tris, 5 mM Pipes, 5 mM MES, 300 mM NaCl, 10% (vol/vol) glycerol, pH 7.0 (AaeR and AaeR-LBD) or pH 6.5 (DDA3937_RS07305) for immediate analysis.

### Differential scanning fluorimetry

Assays were conducted using a MyiQ2 real-time PCR instrument (Bio-Rad), as previously described [140]. Briefly, each 25 μL assay mixture contained 40 μM freshly purified protein dialyzed into analysis buffer, SYPRO Orange (Life Technologies) at 5 × concentration and ligands at final concentrations of 1–2 mM. Ligands from the PM1, PM2A, PM3B, PM4A, and PM5 compound arrays (Fig F in S1 Text) (Biolog, Hayward, CA, USA; https://www.biolog.com/) were dissolved in 50 μL of Milli-Q water, which, according to the manufacturer, corresponds to a concentration of 10–20 mM. Samples were heated from 23 ºC to 85 ºC at a rate of 1 ºC/min. Protein unfolding curves were monitored by detecting changes in SYPRO Orange fluorescence. The thermal denaturation midpoint (Tm) values were determined from the first derivative values of the raw fluorescence data.

### Isothermal titration calorimetry

Measurements were made using a VP-ITC microcalorimeter (MicroCal, Inc., Northampton, MA) at 25 °C. AaeR, AaeR-LBD, and DDA3937_RS07305 were dialyzed into the above described buffers. Proteins at 10–50 μM were placed into the sample cell and titrated with 8.0-12.8 μL aliquots of 1–5 mM ligand solutions made up in the corresponding dialysis buffers. In all cases, the mean enthalpy values measured from ligand injections into the buffer were subtracted from raw titration data prior to data analysis with the ORIGIN software (MicroCal) to determine the corresponding thermodynamic binding parameters.

### Quantification of IAA by gas chromatography–mass spectrometry (GC–MS)

Triplicate cultures of each bacterial strain were inoculated in minimal medium at an initial OD_600_ of 0.075 and incubated at 28 °C in the presence of 0.25 mg/mL L-tryptophan. After 12 h and 24 h, 30 mL culture samples were harvested by centrifugation (7,000 × *g*, 10 min), and the resulting supernatants were filtered through 0.2 µm membranes. In parallel, cell pellets from 30 mL cultures were resuspended in 3 mL minimal medium, lysed using a French press at a gauge pressure of 62.5 lb/in², and centrifuged at 10,000 × *g* for 10 min. Filtered samples from both supernatants and cell lysates were spiked with 5 µg/mL 5-methoxy-indole-3-acetic acid (5-Me-IAA; Merck) as an internal standard, and IAA was extracted as previously described [36]. Briefly, 3 mL of each filtered supernatant was acidified to pH 2.5–3.0 with HCl and extracted three times with 2.5 mL of analytical-grade diethyl ether. The combined ether phases were evaporated to dryness under a gentle stream of N₂. Samples were subsequently reconstituted in 100 µL of BSTFA + TMCS (N,O-bis(trimethylsilyl)trifluoroacetamide + 1% trimethylchlorosilane; Merck) and trimethylsilylated for 1 h at 70 °C. After cooling, 1 µL of each sample was analyzed using a Varian 450 gas chromatograph coupled to a 240 ion trap mass spectrometer, with electron impact ionization (70 eV). GC–MS parameters were as follows: DB-5 column (30 m × 0.25 mm × 0.25 μm film thickness); helium carrier gas at 1 mL/min; injector temperature, 280 °C; oven program, 60 °C (5 min) ramped at 11 °C min ⁻ ¹ to 300 °C; detector temperature, 290 °C. Mass spectra were acquired in full-scan mode (m/z 45–600). IAA and 5-Me-IAA were quantified in SIM mode using m/z 130 and 160, respectively. Peak areas were compared to authentic standards, and IAA concentrations were expressed relative to the wild-type strain after normalization to OD_600_ at 24 h.

### *Solanum tuberosum* virulence and competitive colonization assays

Potato tuber virulence assays were performed as previously described [141]. Briefly, potato tubers (*Solanum tuberosum* (var. Soprano)) were rinsed with sterile distilled water, surface sterilized with 70% (vol/vol) ethanol, and sliced into 15-mm-thick sections. Each slice was inoculated with 5 × 10⁷ CFU of the corresponding *D. dadantii* strains prepared in M9 salts. The inoculated slices were incubated in a growth chamber under controlled conditions (28 ºC, 80% relative humidity) for 48–72 h. Following incubation, tissue maceration was visually assessed. The spatial extent of the macerated area was quantified using ImageJ software [142], and the percentage of maceration was calculated relative to the total slice area. For competition assays, the same protocol and incubation conditions were followed, except that tubers were inoculated with a 5 x 10^7^ CFU 1:1 mixture of *D. dadantii* 3937 and Δ*iaaM*::*km3*. At 72 h post-inoculation (hpi), bacterial populations were quantified by individually sampling 100 mg of tissue from the peripheral region of each macerated slice, homogenizing them in M9 salts, and drop-plating on minimal medium plates with or without 50 μg/mL kanamycin to selectively recover the Δ*iaaM*::*km3* mutant strain. In all cases, at least nine slices were inoculated, and two slices mock-inoculated with sterile M9 salts served as negative controls.

For potato plant virulence assays, *D. dadantii* strains were grown on KB plates at 28 °C for 24 h, resuspended in 10 mM MgCl_2_ and diluted to 5 x 10^7^ CFU/mL. Three-week-old *Solanum tuberosum* (var. Desiree) leaves were syringe-infiltrated with 5 x 10^7^ CFU/mL bacterial suspensions, while three leaves were mock-infiltrated with 10 mM MgCl_2_. Plants were incubated in a growth chamber under controlled conditions (28 ºC, 80% relative humidity, 12-h photoperiod at 100 μE/m^2^ s). At 72 hpi, bacterial populations were determined by sampling five 1 cm^2^ leaf disks per plant. Disks were washed twice with 10 mM MgCl_2_ to eliminate the bacteria from the leaf surface, homogenized, and drop-plated on nutrient broth agar supplemented with the appropriate antibiotics. Bacterial counts from five leaves per plant and three plants per treatment were analyzed across three independent experiments. For competitive colonization assays, experiments were carried out as described above, except that leaves were syringe-infiltrated into the abaxial side with a 5 ⋅ 10^7^ CFU/mL 1:1 mixture of either *D. dadantii* 3937 and Δ*iaaM*::*km3*. Five leaves per plant were inoculated, with three leaves mock-inoculated with 10 mM MgCl_2_ as controls. Plants were incubated in a growth chamber under controlled conditions (28 ºC, 80% relative humidity, 12-h photoperiod at 100 μE/m^2^ s). At 72 hpi, bacterial populations were quantified by sampling five 1 cm² leaf disks per plant. Disks were washed twice with 10 mM MgCl₂, homogenized, and drop-plated on NB agar and NB agar supplemented with 50 μg/mL kanamycin, to selectively recover the Δ*iaaM*::*km3* mutant strain. Bacterial counts from five leaves and three plants per treatment, across three independent experiments, were analyzed.

### Exoenzyme and siderophore assays

*D. dadantii* strains were screened on agar plates for the production of protease, cellulase, pectate lyase, siderophores, beta-glucosidase, lipase, endocellulase, xylanase, phytase, acid and alkaline phosphatase, and lecithinase. For all assays, strains were first grown overnight in LB or minimal medium with glucose as carbon source at 28 °C. Cells were then washed twice with M9 salts and resuspended in the same solution to an OD_600_ of 1. Aliquots (10 µL) were spotted onto the corresponding plates supplemented with 0.25 mg/mL L-tryptophan and incubated at 28 °C for 1–7 days, depending on the enzymatic activity assayed. Media and protocols were used as previously described: Protease [143], pectate lyase and cellulase [144], siderophores [145], beta-glucosidase [146], lipase [147], endo-cellulase and xylanase [148], phytase [149], alkaline and acid phosphatases [150,151], and lecithinase [152]. Enzymatic activities were identified by the formation of characteristic halos around colonies: protease, phytase, and acid phosphatase produced translucent halos; pectate lyase appeared as double cream-colored halos on a translucent blue-green background; cellulase activity produced light blue halos on a dark blue background; β-glucosidase activity was visualized as black halos surrounding colonies; siderophore production appeared as orange halos; lipase and lecithinase activities produced opaque halos; and endocellulase and xylanase activities were indicated by pink and blue halos, respectively.

## Supporting information

S1 Table List of the 774 bacterial taxa possessing gene clusters containing homologous *aaeXAB* genes and their corresponding isolation sources. (XLSX)

S1 Text Supplementary Table A-C, Fig A-M, supplementary materials and methods. (DOCX)

## Abbreviations

PAB: plant-associated bacteria
IAA: indole-3-acetic acid
MIC: minimum inhibitory concentration
LBD: ligand binding domain
IAM: indole-3-acetamide
ITC: isothermal titration calorimetry
*K*_D_: dissociation constant
PCWDE: plant cell wall–degrading enzyme; L-Trp, L-tryptophan
