# Supplementary material for: Auxin biosynthesis and signaling drive virulence and plant adaptation in Dickeya dadantii
Source: PLoS Pathog. 2026 Jul 17;22(7):e1014429. doi: 10.1371/journal.ppat.1014429 (PMC13395407; doi:10.1371/journal.ppat.1014429)
Supplement: S1 Text — (DOCX) [file ppat.1014429.s002.docx]

**Supplementary material to:**

**Auxin biosynthesis and signaling drive virulence and plant adaptation in *Dickeya dadantii***

**Amalia Roca^1,2#^, Saray Santamaría-Hernando^3,4^, Zulema Udaondo^5^, Juan J. Cabrera^6^, Patricia Godoy^6^, Ana Nogueira^6^, Emilia López-Solanilla^3,4^, Miguel A. Matilla^6#^**

^1^Department of Microbiology, Facultad de Farmacia, Campus Universitario de Cartuja, Universidad de Granada, Granada, Spain.

^2^Institute of Biotechnology, Biomedical Research Center (CIBM), University of Granada, Granada, Spain.

^3^Centro de Biotecnología y Genómica de Plantas, Universidad Politécnica de Madrid (UPM)-Instituto Nacional de Investigación y Tecnología Agraria y Alimentaria (INIA-CSIC), Pozuelo de Alarcón, Madrid, Spain.

^4^Department of Biotechnology-Plant Biology, Escuela Técnica Superior de Ingeniería Agronómica, Alimentaria y de Biosistemas, Universidad Politécnica de Madrid (UPM), Madrid, Spain.

^5^Department of Microbial Biotechnology, Centro Nacional de Biotecnología, Consejo Superior de Investigaciones Científicas, Madrid, Spain.

^6^Department of Biotechnology and Environmental Protection, Estación Experimental del Zaidín, Consejo Superior de Investigaciones Científicas, Granada, Spain.

Short title: Auxin signaling in phytopathogenic bacteria

^#^Address correspondence to:

Amalia Roca, [amaliaroca@ugr.es](mailto:amaliaroca@ugr.es); Miguel A. Matilla,[miguel.matilla@eez.csic.es](mailto:miguel.matilla@eez.csic.es)

In this file:

**Table A.** Minimal inhibitory concentration (MIC) values for indole-3-acetic acid (IAA), *p*-hydroxybenzoate (*p*HBA), salicylate and benzoate in *Dickeya dadantii* 3937 strains.

**Table B.** Strains and plasmids used in this study.

**Table C.** Oligonucleotides used in this study.

**Figure A.** Quantification of indole-3-acetic acid (IAA) production by *Dickeya dadantii* 3937 strains measured by gas chromatography coupled to mass spectrometry.

**Figure B.** Impact of *iaaM* mutation on transcript levels of differentially expressed genes identified in the RNA-seq analysis.

**Figure C.** Effect of different concentrations of salicylate and benzoate on the growth kinetics of *Dickeya dadantii* 3937 strains.

**Figure D.** Effect of 1 mM indole-3-acetic acid (IAA) on the growth kinetics of *Dickeya dadantii* 3937 strains.

**Figure E.** The transcriptional regulator AaeR of *Dickeya dadantii* 3937 does not bind indole-3-acetic acid (IAA) or *p*-hydroxybenzoate.

**Figure F.** Composition of the Biolog compound arrays PM1, PM2A, PM3B, PM4A and PM5 used for ligand screening.

**Figure G.** Differential scanning fluorimetry-based thermal shift assays of AaeR-LBD.

**Figure H.** Role of the AaeXAB efflux pump in the virulence of Dickeya dadantii 3937 strains in potato tubers.

**Figure I.** Virulence assays of *D. dadantii* 3937 strains in potato plants.

**Figure J.** Growth of Dickeya dadantii 3937 strains in media mimicking conditions encountered in the leaf environment (A) and potato tubers (B).

**Figure K.** The AaeXAB efflux pump does not contribute to exoenzyme production or siderophore activity in Dickeya dadantii 3937.

**Figure L.** Protein sequence alignment of the MarR-type regulator DDA3937_RS07305 from Dickeya dadantii 3937 with the IAA-binding MarR-type regulators MarR_73 (IadR) from Variovorax paradoxus (A) and IacR from Pseudomonas putida (B).

**Figure M. The *iaaM* mutation does not affect exoenzyme production or siderophore activity in** Dickeya dadantii **3937.**

**Supplementary materials and methods**

**Table A. Minimal inhibitory concentration (MIC) values for indole-3-acetic acid (IAA), *p*-hydroxybenzoate (*p*HBA), salicylate and benzoate in *Dickeya dadantii* 3937 strains.**

| **IAA (mM)** | | ***p*HBA (mM)** | | **Salicylate (mM)** | | **Benzoate (mM)** | |
| --- | --- | --- | --- | --- | --- | --- | --- |
| 3937 wt | ∆*aaeAB* | 3937 wt | ∆*aaeAB* | 3937 wt | ∆*aaeAB* | 3937 wt | ∆*aaeAB* |
| 15 | 4 | 100 | 50 | 4 | 2 | 100 | 100 |

**Table B. Strains and plasmids used in this study.**

| **Strains and plasmids** | **Genotypes or relevant characteristics^a^** | **References** |
| --- | --- | --- |
| **Strains** | | |
| *D. dadantii* 3937 lac- | ∆*lac-*; laboratory strain referred to as wild type | This study |
| *D. dadantii* 3937 ∆*iaaM*::*km3* | ∆*lac-*; Δ*iaaM*::*km3*; Km^R^ | This study |
| *D. dadantii* 3937 ∆*expI*::*km3* | ∆*lac-*; Δ*expI*::*km3*; Km^R^ | This study |
| *D. dadantii* 3937 ∆*vfmI* | ∆*lac-*; Δ*vfmI* | This study |
| *D. dadantii* 3937 ∆*expI* ∆*vfmI* | ∆*lac-*; ∆*expI*::*km3* Δ*vfmI*; Km^R^ | This study |
| *D. dadantii* 3937 ∆*aaeAB*::*km3* | ∆*lac-*; ∆*aaeAB*::*km3*; Km^R^ | This study |
| *D. dadantii* 3937 *tyrR*::*km3* | ∆*lac-*; *tyrR*::*km3*; Km^R^ | This study |
| *D. dadantii* 3937 ∆*trpR*::*km3* | ∆*lac-*; Δ*trpR*::*km3*; Km^R^ | This study |
| *D. dadantii* 3937 ∆*lrhA*::*km3* | ∆*lac-*; Δ*lrhA*::*km3*; Km^R^ | This study |
| *D. dadantii* 3937 ∆*aaeR*::*km3* | ∆*lac-*; Δ*aaeR*::*km3*; Km^R^ | This study |
| *D. dadantii* 3937 ∆*marR_7305*::*km3* | ∆*lac-*; Δ*DDA3937_RS07305*::*km3*; Km^R^ | This study |
| *Escherichia coli* DH5α | F^–^ *endA1* *glnV44* *thi-1*  *recA1*  *relA1*  *gyrA96 deoR* *nupG* *purB20* φ80d*lacZ*ΔM15 Δ(*lacZYA-argF*)U169, hsdR17(*r_K_*^–^*m_K_*^+^), λ^–^ | [1] |
| *E. coli* CC118λ*pir* | *araD*, Δ(*ara*, *leu*), Δ*lacZ74*, *pho*A20, *galK*, *thi-1*, *rspE*, *rpoB*, *argE*, *recA1*, λ*pir* | [2] |
| *E. coli* β2163 | F- RP4-2-Tc::Mu Δ*dapA*::(*erm-pir*); Km^R^, Em^R^ | [3] |
| *E. coli* BL21(DE3) | F^–^ *ompT* *gal* *dcm* *lon* *hsdS_B_*(*r_B_*^–^*m_B_*^–^) λ(DE3 [*lacI* *lacUV5*-*T7p07* *ind1* *sam7* *nin5*]) [*malB*^+^]_K-12_(λ^S^) | [4] |
| **Plasmids** | | |
| pUC18Not | Ap^R^; identical to pUC18 but with two NotI sites flanking pUC18 polylinker | [2] |
| pKNG101 | Sm^R^; *oriR6K mob sacBR* | [5] |
| p34S-km3 | Km^R^, Ap^R^; *km3* antibiotic cassette | [6] |
| pBBR1MCS-5_START | Gm^R^; *oriRK2 mobRK2* | [7] |
| pMAMV392 | Ap^R^; 1.5-kb PCR product generated by overlapping PCR containing a 1.5-kb deletion of *iaaM* of *D. dadantii* 3937 inserted into the EcoRI/HindIII sites of pUC18Not | This study |
| pMAMV393 | Ap^R^, Km^R^; 0.95-kb BamHI fragment containing *km3* cassette of p34S-Km3 was inserted into the BamHI site of *iaaM* in pMAMV392 | This study |
| pMAMV394 | Sm^R^, Km^R^; 2.4-kb NotI fragment of pMAMV393 was cloned at the same site in pKNG101 | This study |
| pMAMV460 | Ap^R^; 1.3-kb PCR product generated by overlapping PCR containing a 1.4-kb deletion of *aaeAB* of *D. dadantii* 3937 inserted into the EcoRI/HindIII sites of pUC18Not | This study |
| pMAMV461 | Ap^R^, Km^R^; 0.95-kb BamHI fragment containing *km3* cassette of p34S-Km3 was inserted into the BamHI site of ∆*aaeAB* of pMAMV460 | This study |
| pMAMV468 | Sm^R^, Km^R^; 2.3-kb NotI fragment of pMAMV461 was cloned at the same site in pKNG101 | This study |
| pMAMV404 | Ap^R^; 1.5-kb PCR product generated by overlapping PCR containing a 2.7-kb deletion of *lacZ* of *D. dadantii* 3937 inserted into the EcoRI/HindIII sites of pUC18Not | This study |
| pMAMV405 | Sm^R^; 1.5-kb NotI fragment of pMAMV404 was cloned at the same site in pKNG101 | This study |
| pUC18-TyrR | Ap^R^; 1.2-kb PCR product containing *tyrR* of *D. dadantii* 3937 inserted into the SacI/HindIII sites of pUC18Not | This study |
| pUC18-TyrR-km3 | Ap^R^, Km^R^; 1-kb PstI fragment containing *km3* cassette of p34S-Km3 was inserted into the PstI site of *tyrR* of pUC18-TyrR | This study |
| pKNG-TyrR | Sm^R^, Km^R^; 2.2-kb NotI fragment of pUC18-TyrR-km3 was cloned at the same site in pKNG101 | This study |
| pUC18-TrpR | Ap^R^; 1.2-kb PCR product generated by overlapping PCR containing a 0.3-kb deletion of *trpR* of *D. dadantii* 3937 inserted into the EcoRI/HindIII sites of pUC18Not | This study |
| pUC18-TrpR-km3 | Ap^R^, Km^R^; 0.95-kb BamHI fragment containing *km3* cassette of p34S-Km3 was inserted into the BamHI site of *trpR* of pUC18-TrpR | This study |
| pKNG-TrpR | Sm^R^, Km^R^; 2.2-kb NotI fragment of pUC18-TrpR-km3 was cloned at the same site in pKNG101 | This study |
| pUC18-LrhA | Ap^R^; 1.6-kb PCR product containing *lrhA* of *D. dadantii* 3937 inserted into the EcoRI/HindIII sites of pUC18Not | This study |
| pUC18-LrhA-km3 | Ap^R^, Km^R^; 1-kb PstI fragment containing *km3* cassette of p34S-Km3 was inserted into the PstI sites within the *lrhA* gene of pUC18-LrhA, thereby deleting a 0.1-kb portion of *lrhA* | This study |
| pKNG-LrhA | Sm^R^, Km^R^; 2.5-kb NotI fragment of pUC18-LrhA-km3 was cloned at the same site in pKNG101 | This study |
| pUC18-AaeR | Ap^R^; 1.5-kb PCR product generated by overlapping PCR containing a 0.7-kb deletion of *aaeR* of *D. dadantii* 3937 inserted into the SacI/HindIII sites of pUC18Not | This study |
| pUC18-AaeR-km3 | Ap^R^, Km^R^; 0.95-kb BamHI fragment containing *km3* cassette of p34S-Km3 was inserted into the BamHI site of *aaeR* in pUC18-AaeR | This study |
| pKNG-AaeR | Sm^R^, Km^R^; 2.5-kb NotI fragment of pUC18-AaeR-km3 was cloned at the same site in pKNG101 | This study |
| pUC18-MarR_7305 | Ap^R^; 1.3-kb PCR product generated by overlapping PCR containing a 0.4-kb deletion of *DDA3937_RS07305* of *D. dadantii* 3937 inserted into the EcoRI/HindIII sites of pUC18Not | This study |
| pUC18-MarR_7305-km3 | Ap^R^, Km^R^; 0.95-kb BamHI fragment containing *km3* cassette of p34S-Km3 was inserted into the BamHI site of *DDA3937_RS07305* in pUC18-MarR_7305 | This study |
| pKNG-MarR_7305 | Sm^R^, Km^R^; 2.3-kb NotI fragment of pUC18-MarR_7305-km3 was cloned at the same site in pKNG101 | This study |
| pMAMV436 | Ap^R^; 1.4-kb PCR product containing a 0.5-kb deletion of *expI* of *D. dadantii* 3937 inserted into the EcoRI/HindIII sites of pUC18Not | This study |
| pMAMV437 | Ap^R^, Km^R^; 0.95-kb BamHI fragment containing *km3* cassette of p34S-Km3 was inserted into the BamHI site of *expI* of pMAMV436 | This study |
| pMAMV438 | Sm^R^, Km^R^; 2.3-kb NotI fragment of pMAMV437 was cloned at the same site in pKNG101 | This study |
| pMAMV227 | Ap^R^; 1.5-kb PCR product containing a 0.9-kb deletion of *vfmI* of *D. dadantii* 3937 inserted into the EcoRI/HindIII sites of pUC18Not | This study |
| pMAMV233 | Sm^R^; 1.5-kb NotI fragment of pMAMV227 was cloned at the same site in pKNG101 | This study |
| pET28b(+) | Km^R^; Protein expression plasmid | Novogene |
| pET28b-AaeR-LBD_3937 | Km^R^; pET28b(+) derivative containing a DNA fragment encoding AaeR-LBD (amino acids 79-309) cloned into the NdeI/HindIII sites | This study^b^ |
| pET28b-AaeR_3937 | Km^R^; pET28b(+) derivative containing a DNA fragment encoding AaeR cloned into the NdeI/BamHI sites | This study |
| pET28b-DDA3937_RS07305 | Km^R^; pET28b(+) derivative containing a DNA fragment encoding DDA3937_RS07305 cloned into the NdeI/HindIII sites | This study |
| pBBR-IaaM | Gm^R^; a 2.3-kb PCR fragment containing the *iaaM* gene cloned into the NdeI/HindIII sites of pBBR1MCS-5_START | This study |
| pBBR-LrhA | Gm^R^; a 1.0-kb PCR fragment containing the *lrhA* gene cloned into the NdeI/HindIII sites of pBBR1MCS-5_START | This study |

^a^Ap, ampicillin; Em, erythromycin; Km, kanamycin; Sm, streptomycin; Gm, gentamicin.

^b^Generated by GenScript.

**Table C. Oligonucleotides used in this study.**

| **Name** | **Sequence (5’-3’)** | **Purpose** | **Reference** |
| --- | --- | --- | --- |
| iaaM-EcoRI-F | taatgaattcgccgctggatgactacacac | Construction of pMAMV392 & mutant verification | This study |
| iaaM-BamHI-R | ggatccGTCATACAGCAGGTCAACACAAGG |  | This study |
| iaaM-BamHI-F | CCTTGTGTTGACCTGCTGTATGACggatccctgaacagtgcctgtgccg |  | This study |
| iaaM-HindIII-R | taataagcttGTGTCCGTTCCGATCGCCAC |  | This study |
| AaeAB-EcoRI-F | taatgaattcgtgctgctgataacgctggc | Construction of pMAMV460 & mutant verification | This study |
| AaeAB-BamHI-R-over | ggatccGCATGCACATGCAGGTTGG |  | This study |
| AaeAB-BamHI-F-over | CCAACCTGCATGTGCATGCGGATCCgctatcaactgctggcgaaagg |  | This study |
| AaeAB-HindIII-R | taataagcttGGTCGACAACGCCGATACC |  | This study |
| 3937-lacZ-EcoRI-F | taatGAATTCggccgcatgatttgccgttg | Construction of pMAMV404 & mutant verification | This study |
| 3937-lacZ-BamHI-R | GGATCCGCATCAGCGTCGGTCAGGTC |  | This study |
| 3937-lacZ-BamHI-F | gacctgaccgacgctgatgcGGATCCcaatctcgacggcttccacatgg |  | This study |
| 3937-lacZ-HindIII-R | taatAAGCTTGTTTCAGCTCCGTGACCGGC |  | This study |
| ExpI-EcoRI-F | taatGAATTCggccgcatgatttgccgttg | Construction of pMAMV436 & mutant verification | This study |
| ExpI-BamHI-R | taatGGATCCGCGTAGTGCAAACACCTCATCC |  | This study |
| ExpI-BamHI-F | taatGGATCCcaacgccgaatccgagcag |  | This study |
| ExpI-HindIII-R | taatAAGCTTCCGACGGACGTTGTGATCATCTC |  | This study |
| VfmI-EcoRI-F | TAATgaattcGTATCCGCCACCCGAATAGC | Construction of pMAMV227 & mutant verification | This study |
| VfmI-BamHI-R | TAAtggatccCCGGCAACTGCATATCGAGA |  | This study |
| VfmI-BamHI-F | TAAtggatccATCTTGCCGTTCAGTCGCTC |  | This study |
| VfmI-HindIII-R | taataagcttGACGTCAGAGTGGCGGAAG |  | This study |
| AaeR_3937-NdeI-F | taatCATatggaacgattaaagagcatgtcg | Construction of pMAMV459 | This study |
| AaeR_3937-BamHI-R | taatggatccCTAAGCCGCTGCGTGATAAG |  | This study |
| RS07305-NdeI-F | taatCATAtgaatgaaaaatccgatgaaatactctacc | Construction of pET28b- DDA3937_RS07305 | This study |
| RS07305-HindIII-R | taataagcttGGAAGCGGTGACCTTTATTGTG |  | This study |
| TyrR.2_3937-SacI-F | TCCTGAGCTCGTAAATCCGCATTTCGGTTGCC | Construction of pUC18-TyrR & mutant verification | This study |
| TyrR.2_3937-HindIII-R | TACTAAGCTTCCGCGTTGCACCAGATCC |  | This study |
| TrpR_3937-EcoRI-F | TAATGAATTCCCGCAGATGAATATCCAGCTTGG | Construction of pUC18-TrpR & mutant verification | This study |
| TrpR_3937-BamHI-R | GGATCCCGGATCATGAAGTGAAGGTGATGTC |  | This study |
| TrpR_3937-BamHI-F | GACATCACCTTCACTTCATGATCCGGGATCCCTACCAGAGCTACCAGAGAAGAAGG |  | This study |
| TrpR_3937-HindIII-R | TAATAAGCTTCTCTGTTTCACCATGGCGAACAAG |  | This study |
| LrhA_3937-EcoRI-F | TAATGAATTCCCGGATTCGGTCTGATGGCTATC | Construction of pUC18-LrhA & mutant verification | This study |
| LrhA_3937-HindIII-R | TAATAAGCTTCCGTGGTGGGAAAATCAACATGG |  | This study |
| RS07305-EcoRI-F | GAATTCgagcgggatcgtcacagcag | pUC18-MarR_7305 & mutant verification | This study |
| RS07305-BamHI-R | ggatccGTGGATAAGCAAGCCAAGCGG |  | This study |
| RS07305-BamHI-F | CCGCTTGGCTTGCTTATCCACggatccggtgatagcgcgccattcctc |  | This study |
| RS07305-HindIII-R | aagcttCTTACTGCGTAGATCCGCCACC |  | This study |
| AaeR-SacI-F | taatgagctcacaacaacctcaaagaccgaaac | pUC18-AaeR & mutant verification | This study |
| AaeR-BamHI-R | ggatccCTGCGGTTGAGCAGTTTCAC |  | This study |
| AaeR-BamHI-F | gtgaaactgctcaaccgcagggatccggtgtgcatcaactacctgacc |  | This study |
| AaeR-HindIII-R | taataagcttGAACTGTGCACGGTGGTGG |  | This study |
| LrhA-Comp-NdeI-F | catatgacaaatacaagtcgccctgtc | Mutant complementation & sequencing | This study |
| LrhA_3937-HindIII-R | TAATAAGCTTCCGTGGTGGGAAAATCAACATGG |  | This study |
| IaaM-NdeI-F-comp | catatgttgactttttggggtggttatatg | Mutant complementation & sequencing | This study |
| IaaM-HindIII-R | taataagcttGTGTCCGTTCCGATCGCCAC |  | This study |
| gyrB_3937-qPCR-F | GACCCGTACGCTCAACAAC | Forward primer for qRT-PCR. *gyrB* (*DDA3937_RS21280*) gene | This study |
| gyrB_3937-qPCR-R | CCACGGCAATTAGCCCTTC | Reverse primer for qRT-PCR. *gyrB* (*DDA3937_RS21280*) gene | This study |
| DDA3937_RS01905-qPCR-F | CACACCTTTGACCGTGTGATTG | Forward primer for qRT-PCR. *iaaM* (*DDA3937_RS01905*) gene | This study |
| DDA3937_RS01905-qPCR-R | ACGCGATATGTCGTGTTCCAG | Reverse primer for qRT-PCR. *iaaM* (*DDA3937_RS01905*) gene | This study |
| DDA3937_RS01910-qPCR-F | CGGATATTCTATTGGCGGTAGC | Forward primer for qRT-PCR. *iaaH* (*DDA3937_RS01910*) gene | This study |
| DDA3937_RS01910-qPCR-R | AAAGAGGCCGGTATTCTTGC | Reverse primer for qRT-PCR. *iaaH* (*DDA3937_RS01910*) gene | This study |
| DDA3937_RS01470-qPCR-F | GAATTCACTCCCGGTCATGG | Forward primer for qRT-PCR. *aaeX* (*DDA3937_RS01470*) gene | This study |
| DDA3937_RS01470-qPCR-R | GATGCAGCAGTCGGATACAG | Reverse primer for qRT-PCR *aaeX* (*DDA3937_RS01470*) gene | This study |
| DDA3937_RS01475-qPCR-F | CCGGCTATCGGGTGGAAATC | Forward primer for qRT-PCR. *aaeA* (*DDA3937_RS01475*) gene | This study |
| DDA3937_RS01475-qPCR-R | TGGTATCGGCGGAGCTACTG | Reverse primer for qRT-PCR. *aaeA* (*DDA3937_RS01475*) gene | This study |
| DDA3937_RS19755-qPCR-F | TGACGGCAGCAAAGACGTG | Forward primer for qRT-PCR. *DDA3937_RS19755* gene | This study |
| DDA3937_RS19755-qPCR-R | TCAGCGGAAGGACCTTTCG | Reverse primer for qRT-PCR. *DDA3937_RS19755* gene | This study |
| DDA3937_RS03055-qPCR-F | GGGTGGAGCAGCTTGGTAG | Forward primer for qRT-PCR. *DDA3937_RS03055* gene | This study |
| DDA3937_RS03055-qPCR-R | GGGCCGGATTTGAACCGAC | Reverse primer for qRT-PCR. *DDA3937_RS03055* gene | This study |

**
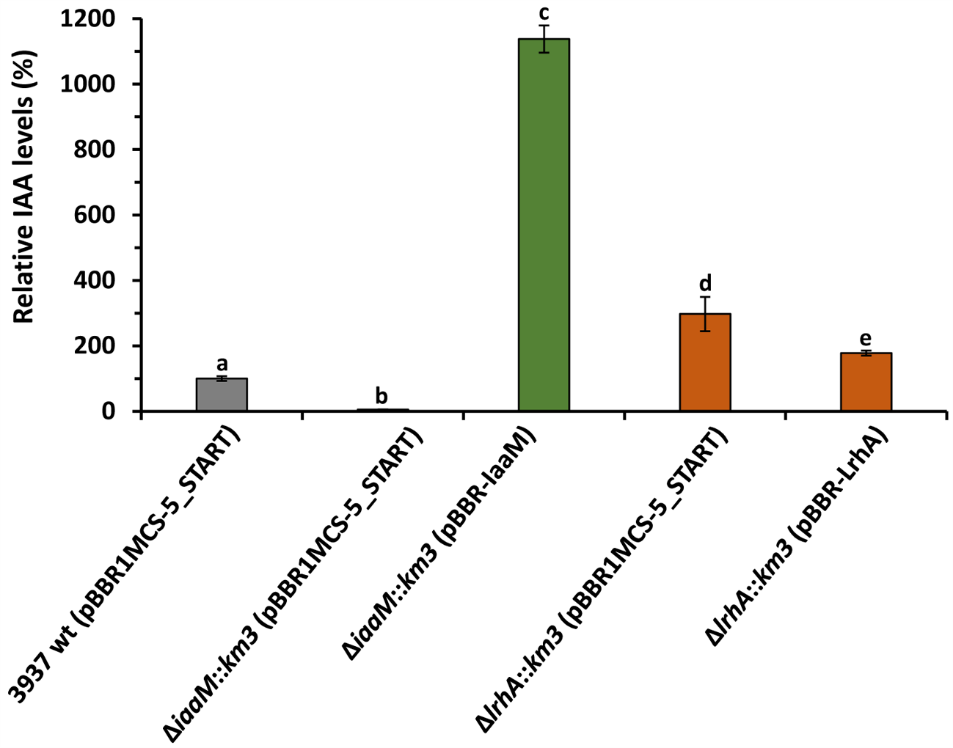
**

**Figure A. Quantification of indole-3-acetic acid (IAA) production by *Dickeya dadantii* 3937 strains measured by gas chromatography coupled to mass spectrometry.** Shown are the relative IAA levels in supernatants of different *D. dadantii* 3937 strains grown at 28 °C for 24 h in minimal medium supplemented with 0.25 mg/mL L-Trp. Data represent means and standard errors of three biological replicates. Unpaired t-test, different letters indicate that data are statistically different, p < 0.05.

**
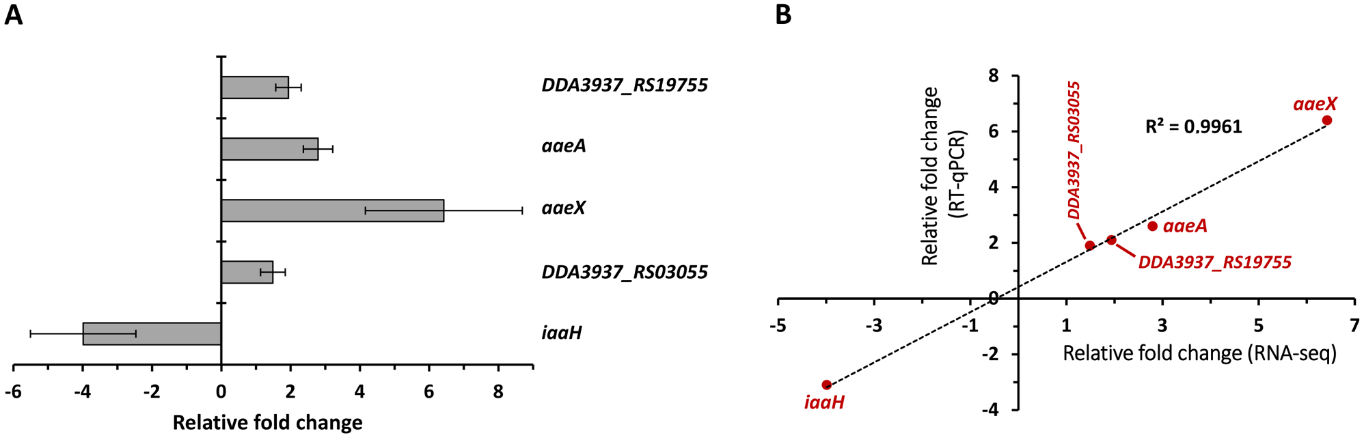
**

**Figure B. Impact of *iaaM* mutation on transcript levels of differentially expressed genes identified in the RNA-seq analysis. A,** Shown are the fold-change mRNA levels of selected genes in the wild-type compared with the Δ*iaaM*::*km3* mutant strains as measured by quantitative RT-PCR (RT-qPCR) under the same conditions used for the RNA-seq analysis. Data are the means and standard deviations of three biological replicates, each conducted in triplicate. **B**, Correlation between transcript levels quantified by RNA-seq and RT-qPCR. The regression line and the coefficient of determination (R^2^) are shown.


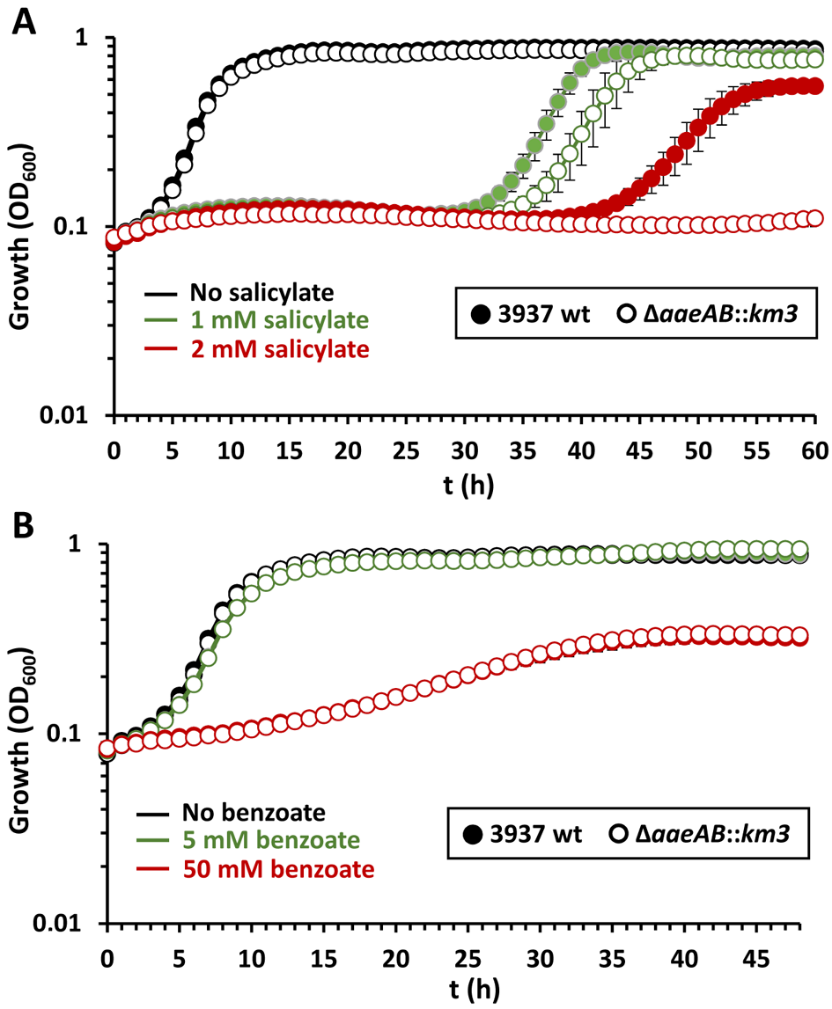


**Figure C. Effect of different concentrations of salicylate and benzoate on the growth kinetics of *Dickeya dadantii* 3937 strains.** Shown is the growth of *D. dadantii* 3937 strains in minimal medium in the absence of presence of different concentrations of salicylate (**A**) and benzoate (**B**). The bioassays were repeated at least three times and representative results of one biological replicate are shown. Data are means and standard deviations of five technical replicates. Some standard deviations are minor and are not visible in the corresponding growth curves. Growth was measured using Bioscreen Microbiological Growth Analyser (Oy Growth Curves Ab Ltd, Helsinki, Finland).

**
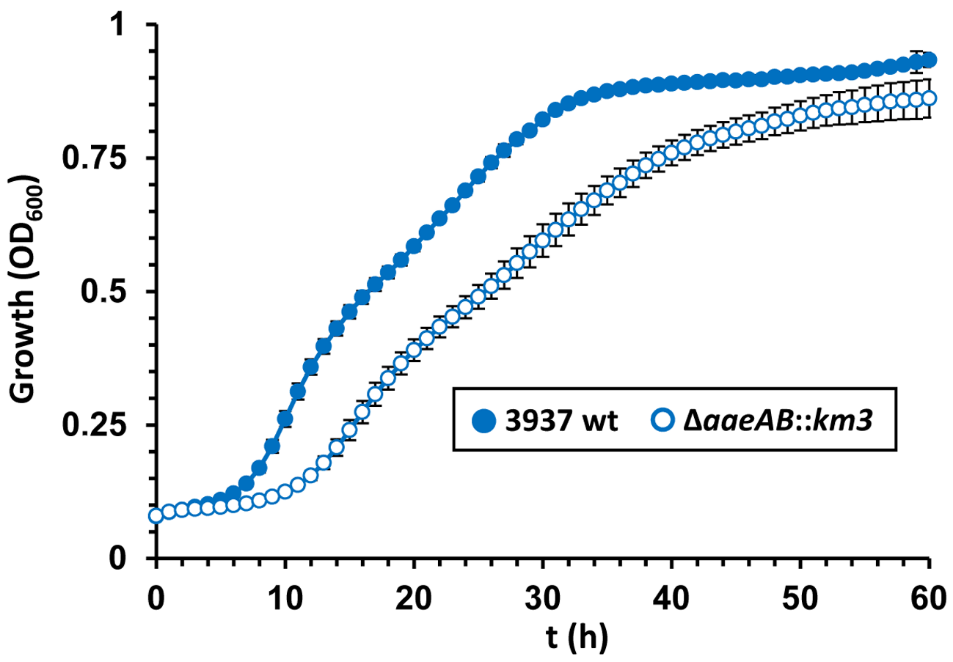
**

**Figure D. Effect of 1 mM indole-3-acetic acid (IAA) on the growth kinetics of *Dickeya dadantii* 3937 strains.** Shown is the growth of *D. dadantii* 3937 strains in minimal medium in the presence of 1 mM IAA. The bioassays were repeated at least three times and representative results of one biological replicate are shown. Data are means and standard deviations of five technical replicates. Some standard deviations are minor and are not visible in the corresponding growth curves. Growth was measured using Bioscreen Microbiological Growth Analyser (Oy Growth Curves Ab Ltd, Helsinki, Finland).

**
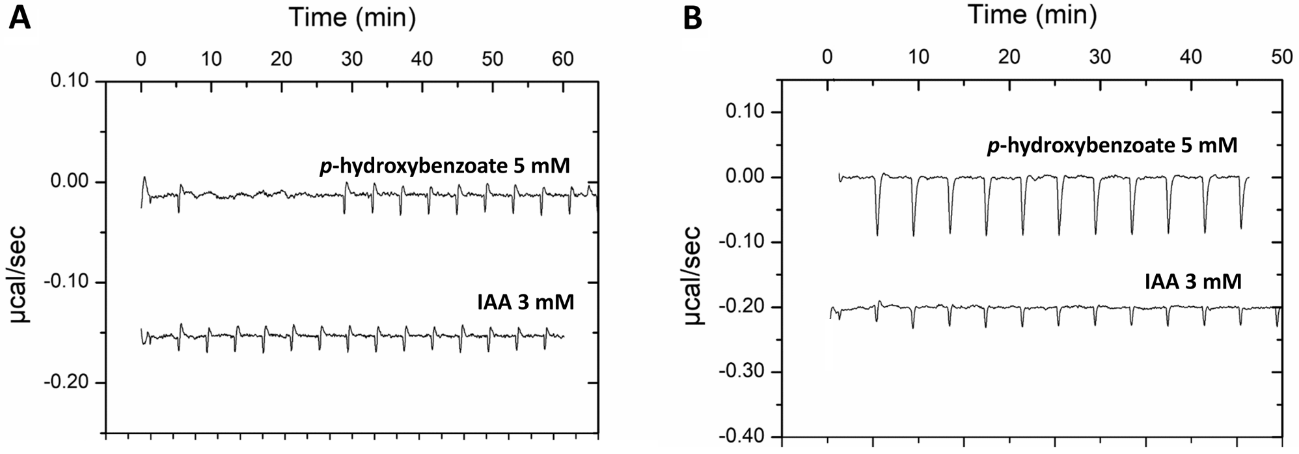
**

**Figure E. The transcriptional regulator AaeR of *Dickeya dadantii* 3937 does not bind indole-3-acetic acid (IAA) or *p*-hydroxybenzoate.** Shown are isothermal titration calorimetry analyses of AaeR-LBD (**A**) and full-length AaeR (**B**). Shown are the raw data for the titration of 50 μM of AaeR-LBD or AaeR with 12.8 μL aliquots of IAA (3 mM) and *p*-hydroxybenzoate (5 mM) solutions.

**Figure F. Composition of the Biolog compound arrays PM1, PM2A, PM3B, PM4A and PM5 used for ligand screening.**

**
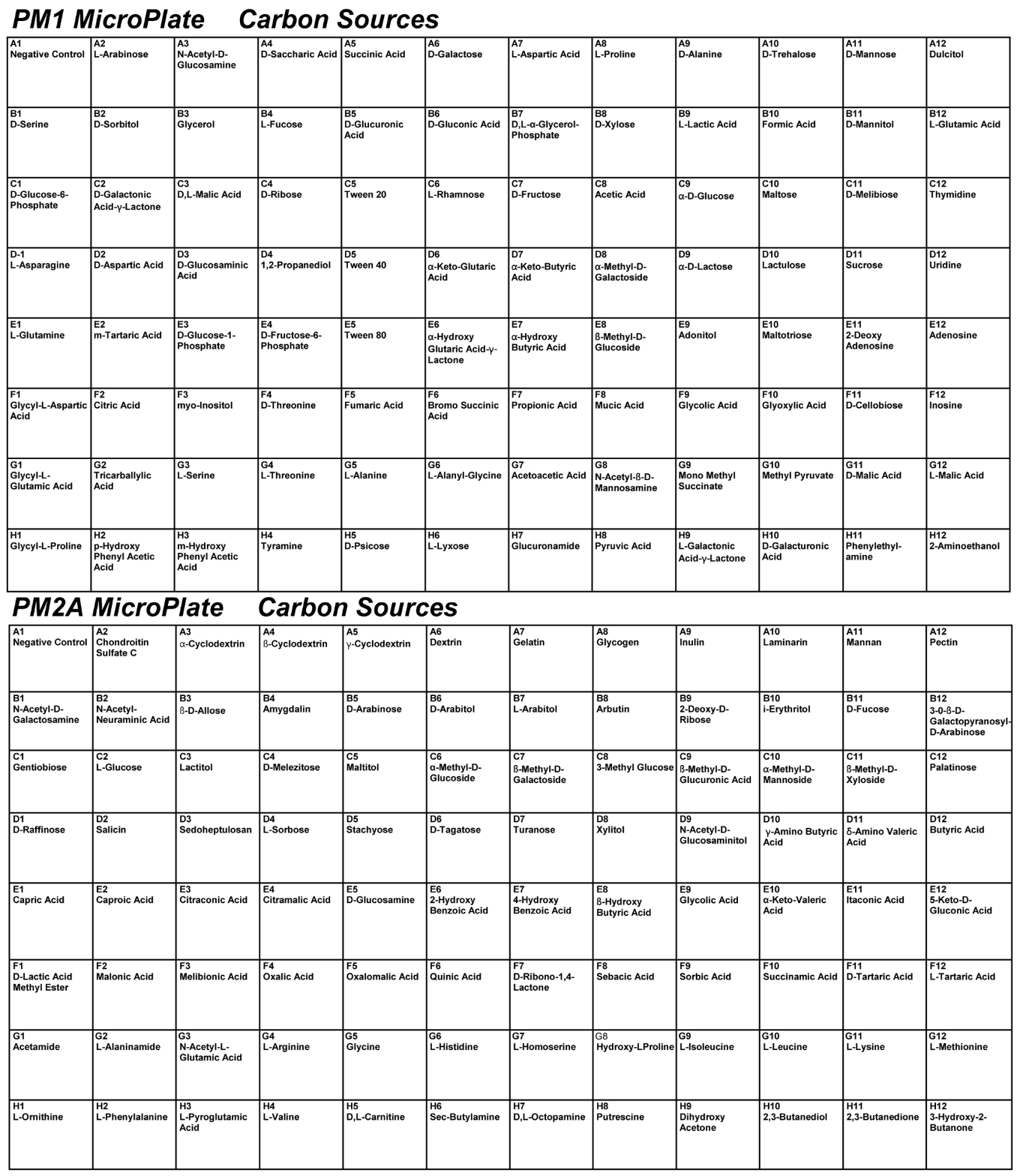
**

**
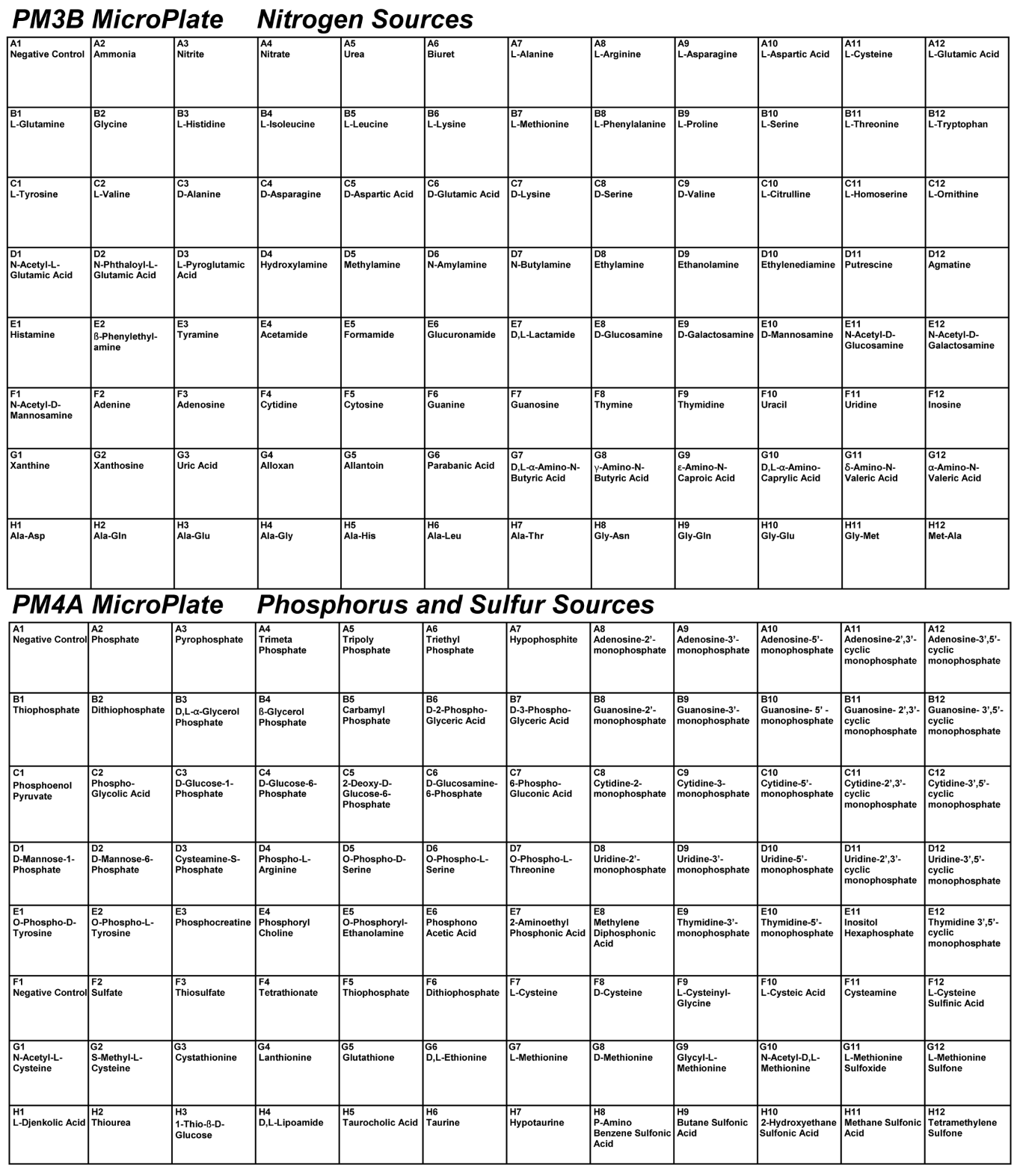
**

**
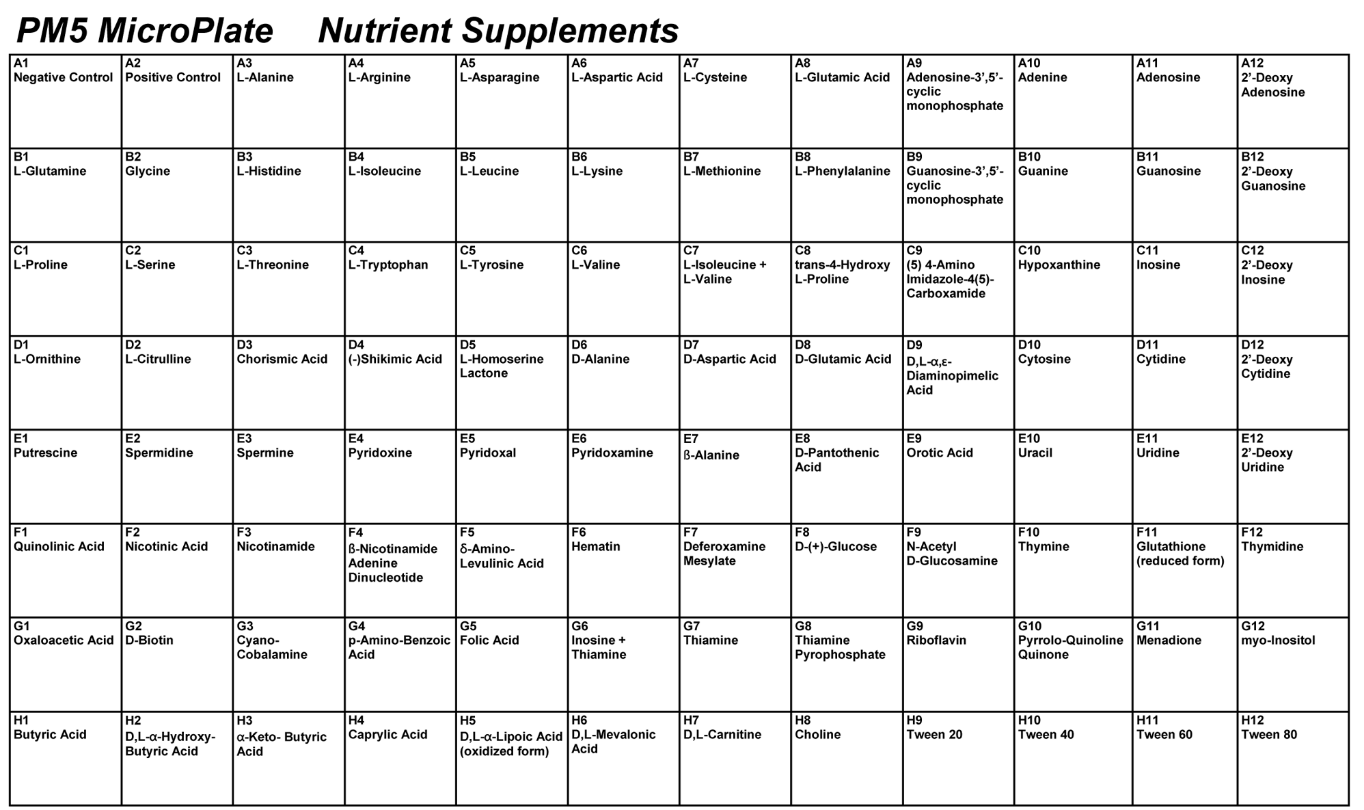
**


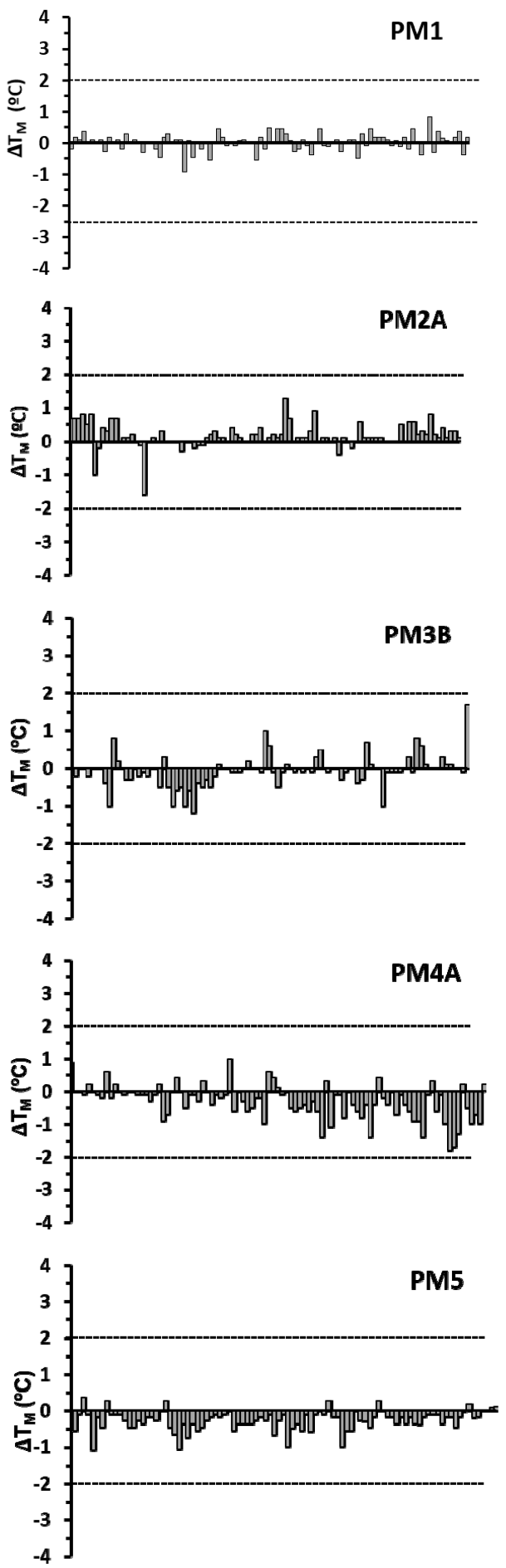


**Figure G. Differential scanning fluorimetry-based thermal shift assays of AaeR-LBD.** Shown are the changes in the midpoint of protein unfolding transitions (Tm) caused by the presence of different compounds from the Biolog Phenotype MicroArray plates PM1, PM2A, PM3B, PM4A, and PM5, relative to the ligand-free protein.

**
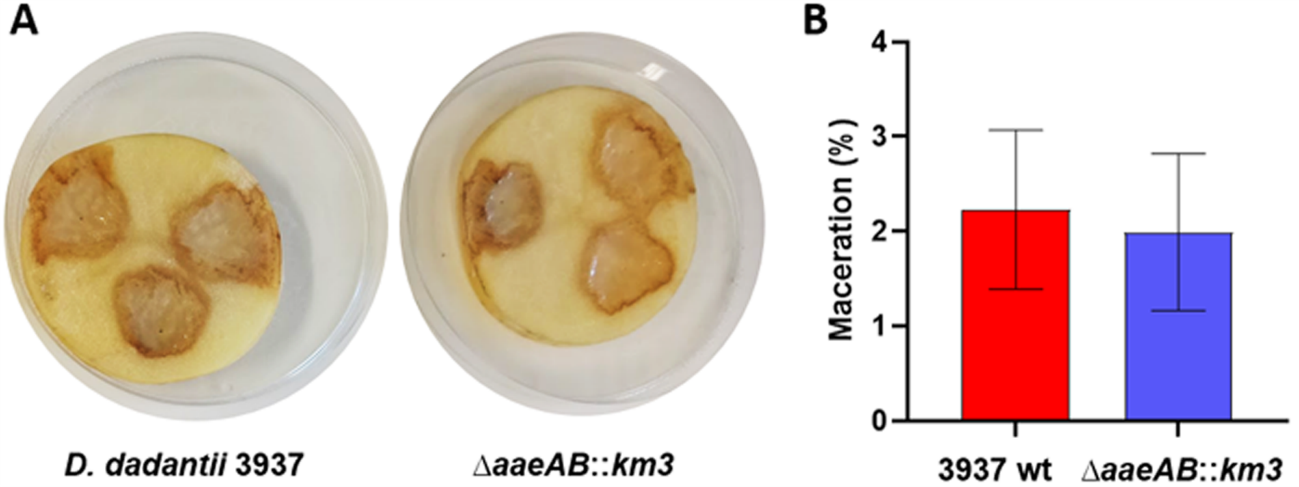
**

**Figure H. Role of the AaeXAB efflux pump in the virulence of Dickeya dadantii 3937 strains in potato tubers. A**, Representative images of tissue maceration caused by D. dadantii strains after 72 h of incubation at 28 °C. Bioassays were repeated at least three times, and representative results are shown. **B**, Quantification of maceration in potato tubers shown in (A). The macerated area was measured using ImageJ software [8], and the percentage of maceration was calculated relative to the total slice area. Data represent means and standard deviations from at least nine technical replicates.


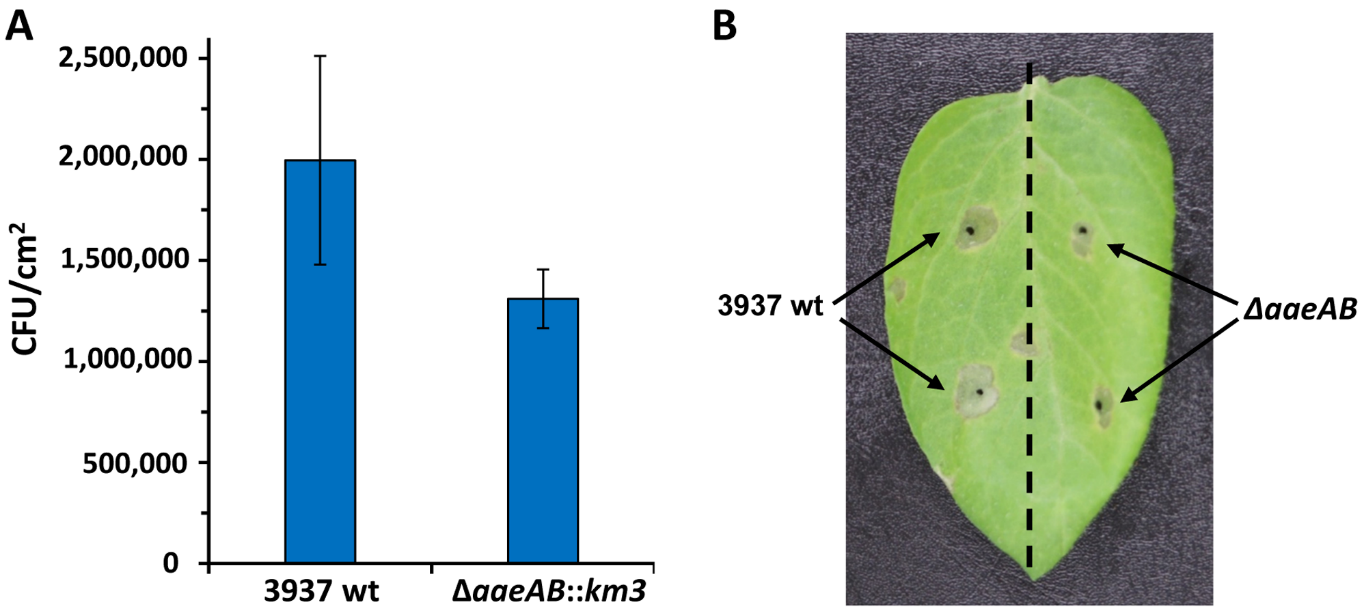


**Figure I. Virulence assays of *D. dadantii* 3937 strains in potato plants. A,** Virulence of *D. dadantii* 3937 strains in potato leaves, expressed in CFU/cm^2^. Virulence was assessed based on bacterial population sizes in potato leaves at 72 h after syringe-infiltration (5·10^7^ CFU/mL). Data were corrected based on the initial inoculum and are means and standard errors from three independent experiments conducted in triplicate. No statistically significant differences were observed. **B**, Representative image of disease symptoms observed 72 h after inoculation in the virulence assays shown in panel (A).


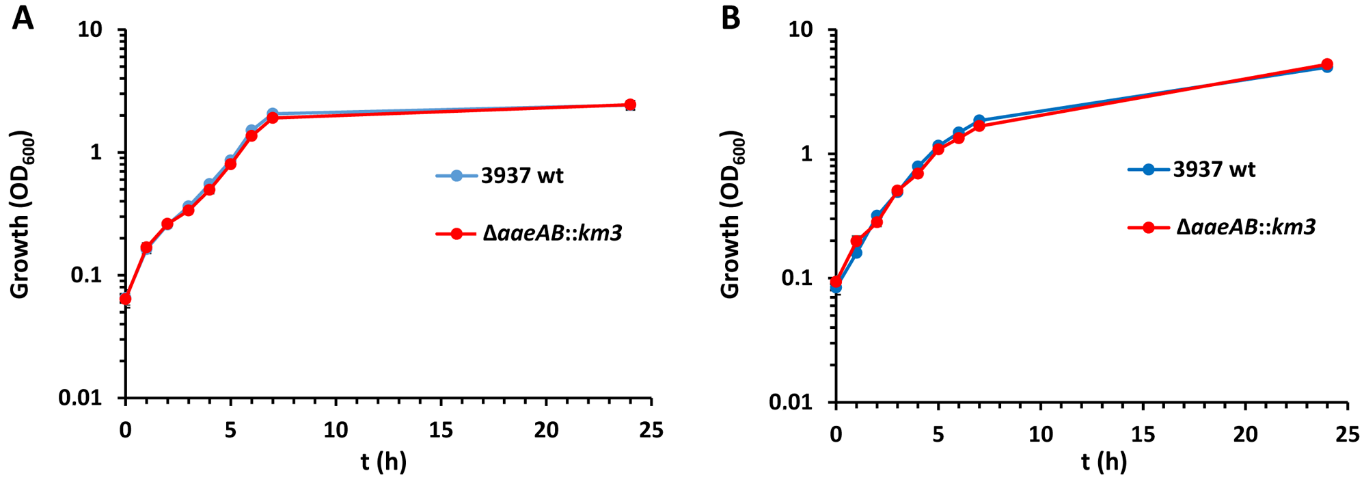


**Figure J. Growth of Dickeya dadantii 3937 strains in media mimicking conditions encountered in the leaf environment (A) and potato tubers (B).** (**A**) Growth of D. dadantii strains in M63 minimal salt medium supplemented with 0.2% (w/vol) sucrose and 0.2% (w/vol) polygalacturonic acid. (**B**) Growth of D. dadantii strains in potato dextrose broth. In all cases, data represent the mean ± standard deviation of two independent experiments, each including three technical replicates per strain. Some standard deviations are minor and are not visible in the corresponding growth curves.

**
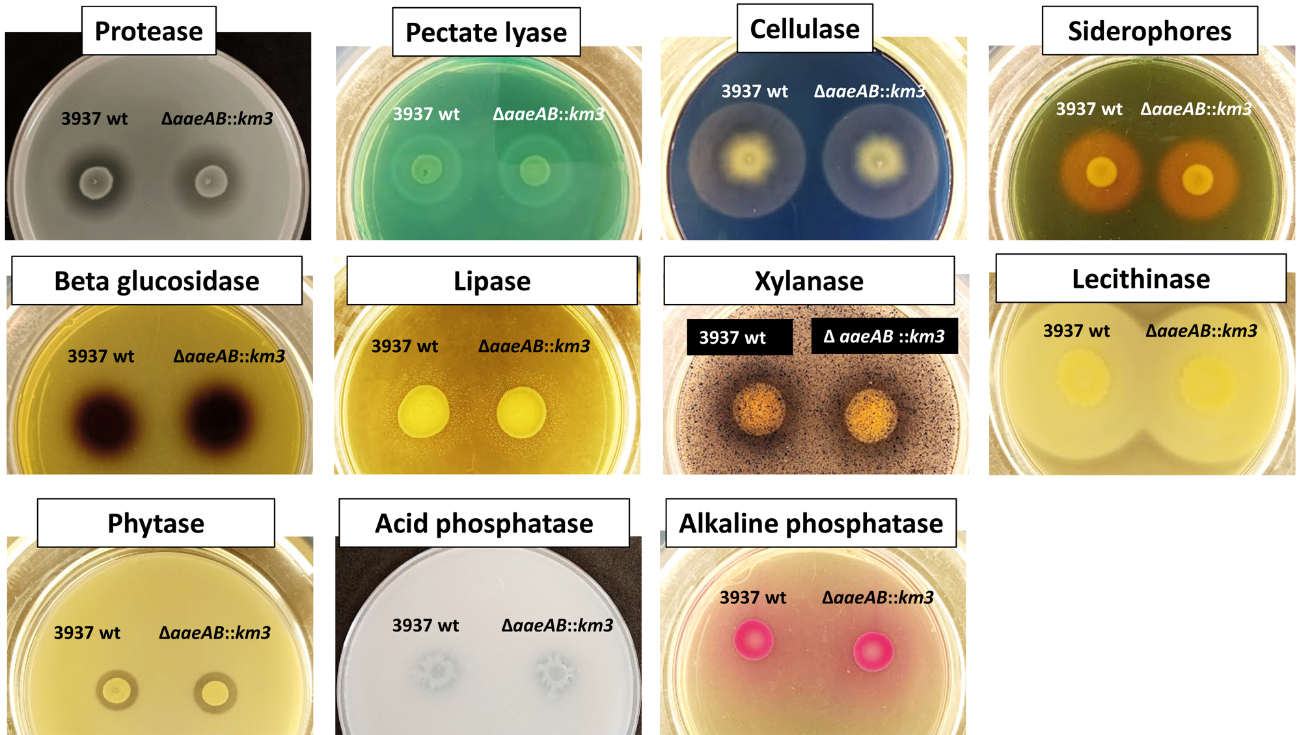
**

**Figure K. The AaeXAB efflux pump does not contribute to exoenzyme production or siderophore activity in Dickeya dadantii 3937.** On each plate, enzymatic activity or siderophore production is indicated by the presence of a halo surrounding the bacterial colony or by a color change to dark brown (β-glucosidase) or pink (alkaline phosphatase). Bioassays were performed at least three times, and representative results from one biological replicate are shown.

**A**

MarR_73 1 MAEQPPETHRFVDDYLPALLAQASQLISSEFHEVARQHGFSVSEWRVMAS 50

|.|:..| :...|..|:...:|...:..::.......:..:::|:.|

DDA3937_RS07305 1 MNEKSDE----ILYPLGLLIHLVNQFKDNLLNDYFADSDITAPQFKVLMS 46

MarR_73 51 LAGSEPISIGQLAQVTVTKQPTVTRLLDRMEARGQVERLPHESDRRITLV 100

:.......: ::::..:.....::|:::||..|..:.||||.||:|..::

DDA3937_RS07305 47 IYKGFTSPV-EVSKNVMMDGGALSRMIERMVKRELLLRLPHPSDKRQVIL 95

MarR_73 101 RITRKGLKAVEHLMELAREHERRVLEPFGLR----RAEELKQTLRQMIDL 146

.:|.|||.. |.:..||..|.||.....| ..|:|.|.|.:|:..

DDA3937_RS07305 96 ALTEKGLAI---LQQFEREGMRIVLSQTTARLTYQEVEQLMQLLIKMLPD 142

MarR_73 147 HVHVPVEE--PEED 158

.|...... ....

DDA3937_RS07305 143 EVIARHSSHLTYPQ 156

**B**

IacR 1 MSNAKNTSAASPARKGHSHHDPASDEFRKEDFPFYWLARVHGRYTQNM-E 49

|:.. |||.. :|...|..:..::..|: .

DDA3937_RS07305 1 MNEK-------------------SDEIL---YPLGLLIHLVNQFKDNLLN 28

IacR 50 RLLKKIDLDVPRWRVLWILNENGESSISEISTHAIAKLSTITKIVYRMKE 99

......|:..|:::||..:.: |.:|..|:|.:.:.....:::::.||.:

DDA3937_RS07305 29 DYFADSDITAPQFKVLMSIYK-GFTSPVEVSKNVMMDGGALSRMIERMVK 77

IacR 100 DGLVDTAPSPEDGRVTQVRITEVGLQNIERMQ-EVTRELFQRSFKGLTEA 148

..|:...|.|.|.|...:.:||.||..:::.: |..|.:..::...||..

DDA3937_RS07305 78 RELLLRLPHPSDKRQVILALTEKGLAILQQFEREGMRIVLSQTTARLTYQ 127

IacR 149 QVQRLNRML------EVVFHNLETL---- 167

:|::|.::| ||:..:...|

DDA3937_RS07305 128 EVEQLMQLLIKMLPDEVIARHSSHLTYPQ 156

**Figure L. Protein sequence alignment of the MarR-type regulator DDA3937_RS07305 from Dickeya dadantii 3937 with the IAA-binding MarR-type regulators MarR_73 (IadR) from Variovorax paradoxus (A) and IacR from Pseudomonas putida (B).** Key IAA-binding residues - Tyr15, Ala18, Ser28, His32, Trp45, Arg46, and Val65 in IadR, and Phe27, Tyr35, Tyr44, Trp62, Arg63, Trp66 in IacR - are highlighted in yellow. Residues implicated in IAA binding were defined by [9].

**
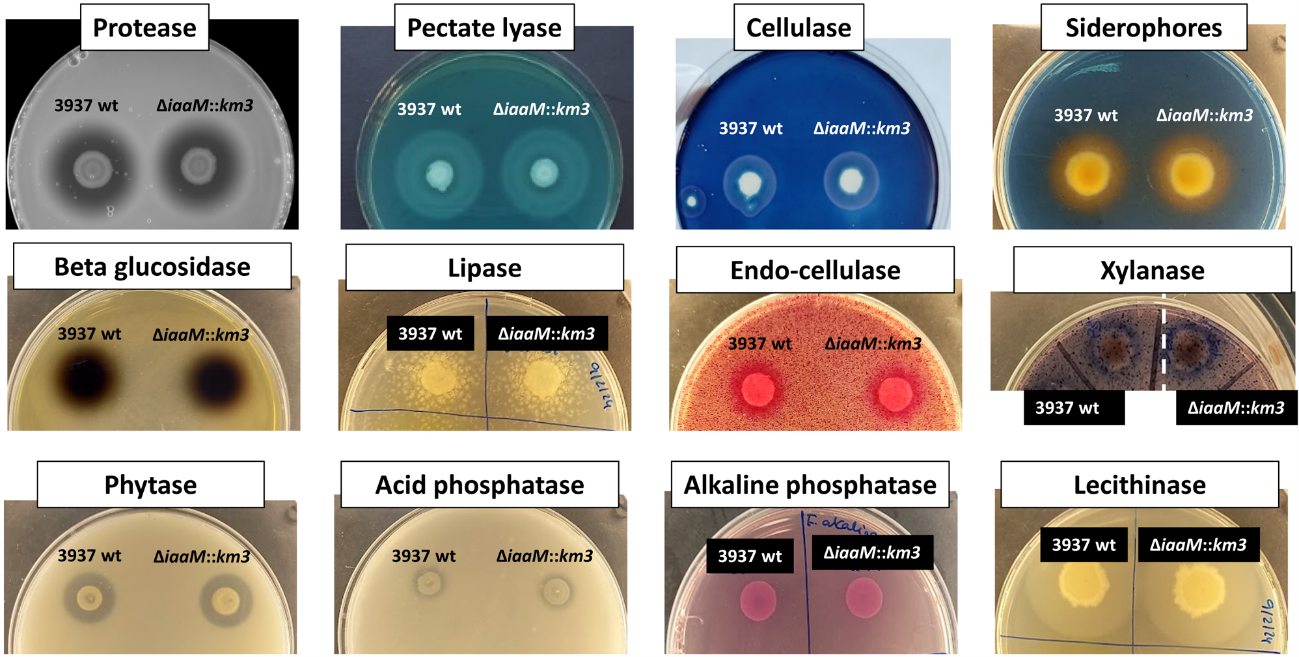
**

**Figure M. The *iaaM* mutation does not affect exoenzyme production or siderophore activity in Dickeya dadantii 3937. O**n each plate, enzymatic activity or siderophore production is indicated by the presence of a halo surrounding the bacterial colony or by a color change to dark brown (β-glucosidase) or pink (alkaline phosphatase). Bioassays were performed at least three times, and representative results from one biological replicate are shown.

**Supplementary materials and methods**

**Identification of regulators associated with AaeABX-Like efflux pump systems**

To identify candidate AaeR regulators associated with the AaeXAB efflux pump clusters, we queried UniProt using two complementary criteria: proteins annotated as ‘HTH transcriptional activator’, ‘LysR family transcriptional regulator’, and proteins containing both PF00126 (Bacterial regulatory helix-turn-helix protein, lysR family; HTH_1) and PF03466 (LysR substrate binding domain; LysR_substrate), which correspond to the Pfam domains present in the AaeR HTH-type transcriptional activator of *Escherichia coli*. After removing redundant entries, this search yielded 1,195,832 unique proteins. Candidate AaeR proteins were then compared against our curated AaeXAB dataset using ORF/locus identifiers and taxonomic information. Briefly, AaeA, AaeB, and AaeX hits were first grouped into putative subclusters within each genome by parsing ordered locus tags and separating loci that were more than 5 ORFs apart. Candidate AaeR proteins were then mapped to the same genome prefix and taxonomic identifier, and their relative position with respect to each AaeXAB subcluster was estimated from the ordered locus numbers. For each AaeABX subcluster, the closest candidate regulator was retained as the best hit.

**References**

1. Woodcock DM, Crowther PJ, Doherty J, Jefferson S, DeCruz E, Noyer-Weidner M, et al. Quantitative evaluation of *Escherichia coli* host strains for tolerance to cytosine methylation in plasmid and phage recombinants. Nucleic Acids Res. 1989;17: 3469–3478. doi: 10.1093/nar/17.9.3469

2. Herrero M, de Lorenzo V, Timmis KN. Transposon vectors containing non-antibiotic resistance selection markers for cloning and stable chromosomal insertion of foreign genes in gram-negative bacteria. J Bacteriol. 1990;172: 6557–6567. doi: 10.1128/jb.172.11.6557-6567.1990

3. Demarre G, Guerout AM, Matsumoto-Mashimo C, Rowe-Magnus DA, Marliere P, Mazel D. A new family of mobilizable suicide plasmids based on broad host range R388 plasmid (IncW) and RP4 plasmid (IncPalpha) conjugative machineries and their cognate *Escherichia coli* host strains. Res Microbiol. 2005;156: 245–255. doi:10.1016/j.resmic.2004.09.007

4. Jeong H, Barbe V, Lee CH, Vallenet D, Yu DS, Choi SH, et al. Genome sequences of *Escherichia coli* B strains REL606 and BL21(DE3). J Mol Biol. 2009;394: 644–652. doi:10.1016/j.jmb.2009.09.052

5. Kaniga K, Delor I, Cornelis GR. A wide-host-range suicide vector for improving reverse genetics in gram-negative bacteria: inactivation of the *blaA* gene of *Yersinia enterocolitica*. Gene. 1991;109: 137–141. doi: 10.1016/0378-1119(91)90599-7

6. Dennis JJ, Zylstra GJ. Plasposons: modular self-cloning minitransposon derivatives for rapid genetic analysis of gram-negative bacterial genomes. Appl Environ Microbiol. 1998;64: 2710–2715. doi: 10.1128/AEM.64.7.2710-2715.1998

7. Obranic S, Babic F, Maravic-Vlahovicek G. Improvement of pBBR1MCS plasmids, a very useful series of broad-host-range cloning vectors. Plasmid. 2013;70: 263–267. doi:10.1016/j.plasmid.2013.04.001

8. Schneider CA, Rasband WS, Eliceiri KW. NIH Image to ImageJ: 25 years of image analysis. Nat Methods. 2012;9: 671–675. doi:10.1038/nmeth.2089

9. Conway JM, Walton WG, Salas-González I, Law TF, Lindberg CA, Crook LE, et al. Diverse MarR bacterial regulators of auxin catabolism in the plant microbiome. Nat Microbiol. 2022;7: 1817–1833. doi:10.1038/s41564-022-01244-3
